# Photocathodes beyond NiO: charge transfer dynamics in a π-conjugated polymer functionalized with Ru photosensitizers

**DOI:** 10.1038/s41598-021-82395-x

**Published:** 2021-02-02

**Authors:** Ruri A. Wahyuono, Bianca Seidler, Sebastian Bold, Andrea Dellith, Jan Dellith, Johannes Ahner, Pascal Wintergerst, Grace Lowe, Martin D. Hager, Maria Wächtler, Carsten Streb, Ulrich S. Schubert, Sven Rau, Benjamin Dietzek

**Affiliations:** 1 Department Functional Interfaces, Leibniz Institute of Photonic Technology (IPHT) Jena e.V., Albert-Einstein-Strasse 9, 07745 Jena, Germany; 2 Institute of Physical Chemistry, Friedrich Schiller University Jena, Helmholtzweg 4, 07743 Jena, Germany; 3 Department of Engineering Physics, Institut Teknologi Sepuluh Nopember, Jl. Arief Rahman Hakim, Surabaya, 60111 Indonesia; 4 Institute of Inorganic Chemistry I, Ulm University, Albert-Einstein-Allee 11, 89081 Ulm, Germany; 5 Univ. Grenoble Alpes, CNRS, CEA, IRIG, Laboratoire de Chimie et Biologie des Métaux, 17 rue des Martyrs, 38000 Grenoble, France; 6 Laboratory of Organic and Macromolecular Chemistry (IOMC), Friedrich Schiller University Jena, Humboldtstrasse 10, 07743 Jena, Germany; 7 Center for Energy and Environmental Chemistry Jena (CEEC Jena), Friedrich Schiller University Jena, Philosophenweg 7a, 07743 Jena, Germany

**Keywords:** Photochemistry, Physical chemistry, Polymer chemistry

## Abstract

A conductive polymer (poly(*p*-phenylenevinylene), **PPV**) was covalently modified with Ru^II^ complexes to develop an all-polymer photocathode as a conceptual alternative to dye-sensitized NiO, which is the current state-of-the-art photocathode in solar fuels research. Photocathodes require efficient light-induced charge-transfer processes and we investigated these processes within our photocathodes using spectroscopic and spectro-electrochemical techniques. Ultrafast hole-injection dynamics in the polymer were investigated by transient absorption spectroscopy and charge transfer at the electrode–electrolyte interface was examined with chopped-light chronoamperometry. Light-induced hole injection from the photosensitizers into the PPV backbone was observed within 10 ps and the resulting charge-separated state (CSS) recombined within ~ 5 ns. This is comparable to CSS lifetimes of conventional NiO-photocathodes. Chopped-light chronoamperometry indicates enhanced charge-transfer at the electrode–electrolyte interface upon sensitization of the PPV with the Ru^II^ complexes and p-type behavior of the photocathode. The results presented here show that the polymer backbone behaves like classical molecularly sensitized NiO photocathodes and operates as a hole accepting semiconductor. This in turn demonstrates the feasibility of all-polymer photocathodes for application in solar energy conversion.

## Introduction

Artificial photosynthesis is a promising pathway for solar-fuel production^1–5^. One way of realizing an artificial photosynthetic system is to graft redox-active photosensitizers and catalysts to semiconductor electrodes to give dye-sensitized photoelectrochemical cells^3–6^. Ru^II^ poly-pyridyl complexes are widely used as chromophores for dye-sensitized electrodes. They exhibit high absorptivity in the visible region and redox properties that are easily tuned by ligand modification^1–16^. In the case of Ru^II^ polypyridyl complex-sensitized photocathodes for hydrogen generation, the semiconductor electrodes play the role of electron donor while the chromophores act as light harvesting and charge-separating units^7–12^. While metal oxide-based semiconducting electrodes such as NiO have attracted widespread interest^13^, the tuning of their physicochemical properties by chemical modification is difficult. Recently, hole conducting organic polymers have been put forward as viable alternatives for metal oxide semiconductors. This materials class has already received enormous interest as semiconducting organic components in organic photovoltaics (OPVs)^14–16^. A few pioneering studies have introduced such polymer-chromophore systems as light harvesters in artificial photosynthesis and have investigated their energy transfer properties in proof-of-principle studies^17,18^.

For photoinduced proton reduction Suzuki et al.^17–20^ developed water-insoluble polymer-photosensitizer films, in which the Ru^II^ polypyridyl complexes were coordinated to imidazolyl residues on a partially quarternized poly(1-vinylimidazole) backbone. Schanze and co-workers^21–23^ reported on polystyrenes containing [Ru(bpy)_3_]^2+^ (bpy = 2,2′-bipyridine) as covalently attached pendant chromophores and [Ru(tpy)(phenq)]^2+^ (tpy = 2,2′;6,2″-terpyridine, phenq = 2-(quinol-8′-yl)-1,10-phenanthroline) as catalytic centers for photocatalytic water oxidation^24,25^. However, these studies did not exploit the electronic properties of the polymer as it acted merely as a support; charge transfer from the chromophores to a sacrificial donor and a metal oxide semiconductor anode material was required for photocatalytic proton reduction^17–20^ and water oxidation^23^, respectively. Furthermore, covalent integration of metal complexes in poly(*p*-phenylenevinylene) (**PPV**) derivatives has been established and the applicability of the resulting materials, *e.g.,* in solar fuel conversion has been indicated^26–28^: Yu and co-workers developed **PPV** functionalized with polypyridyl-based Ru^II^ complexes by direct integration of the Ru-bound bipyridyl units into the π-conjugated main chain of the **PPV**^26–28^.

Inspired by Schanze’s report on transition metal complexes integrated into polystyrene^21–23^, we propose the π-conjugated **PPV** as an appealing alternative hole-transport matrix for metal-oxide free photocathodes for light-driven water splitting. Using polymers such as **PPV** as both a structural support and charge transport matrix utilizes the full capability of these materials. Functionalized **PPV** derivatives, particularly poly[2-methoxy-5-(2′-ethyl-hexoxy)-1,4-phenylenevinylene] (MEH-PPV), are frequently used in polymer solar cells^14–16,29,30^. Importantly, their HOMO level (ca. 4.8–4.9 eV)^31,32^ is comparable to the valence band of p-type NiO (ca. 5.0–5.1 eV)^33,34^. Therefore, “molecularly sensitized **PPV**” could replace semiconductor electrodes in photoelectrochemical cells if the charge-transfer dynamics of the system are suitable for coupling with catalytic hydrogen evolution processes.

Herein, we report first steps towards all-polymer photocathodes for artificial photosynthesis by facile covalent attachment of Ru complexes as side-chains in **PPV** polymers via CLICK chemistry. The analysis of the charge transfer dynamics of these complexes reveals their suitability for visible light harvesting, charge separation and hole injection into the **PPV**.

## Results and discussion

### Synthesis of PPV-Ru

**Figure 1:**
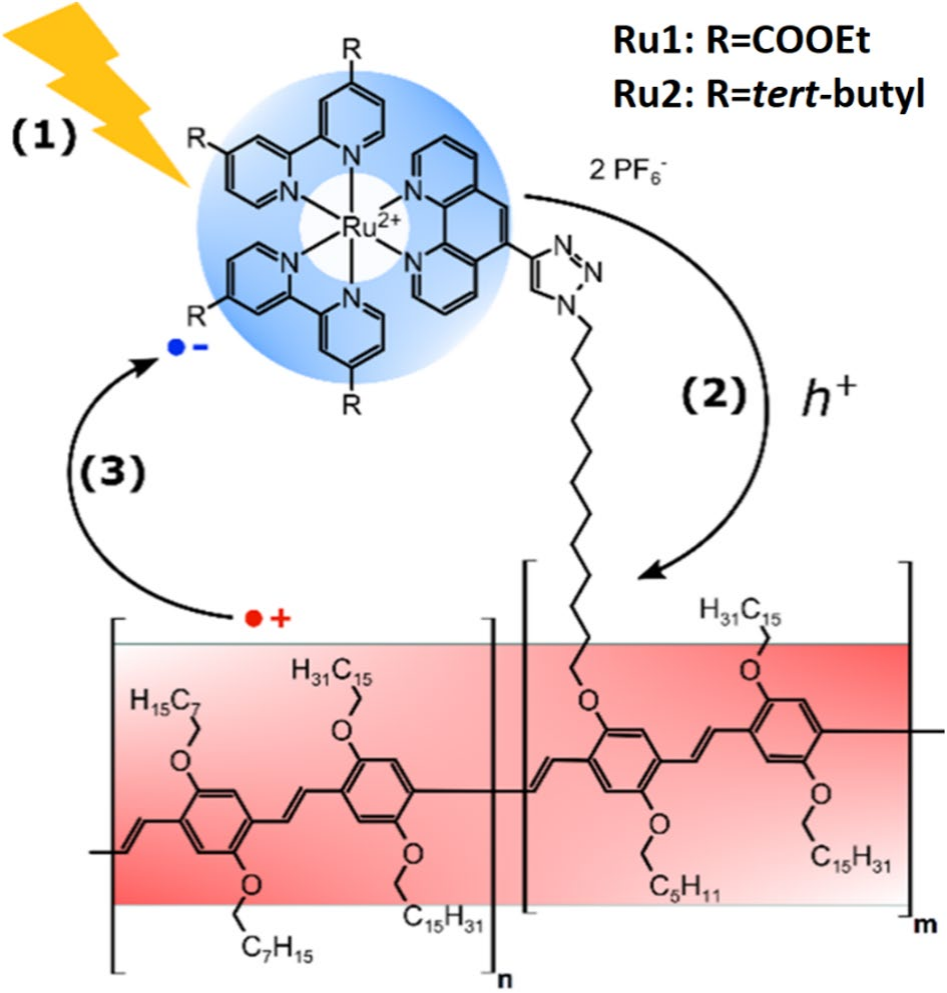
Schematic representation of the molecular structures of π-conjugated **PPV** with Ru^II^ chromophores in the side chains, i.e. **Ru1** and **Ru2**. Transient absorption spectroscopy is employed to unravel light-driven (1) hole injection (2) and charge recombination (3) dynamics in **PPV-Ru**. The red and blue shadings schematically indicate photogenerated holes residing on the **PPV** and the radical anion after hole injection from the photosensitizer to the **PPV**, respectively.

We have developed a synthetic route towards two prototype systems, **PPV-Ru1** and **PPV-Ru2**, where Ru^II^-polypyridyl complexes were linked covalently to the **PPV** backbone by CLICK chemistry (the details of the synthesis and preparation of **PPV-Ru1** and **PPV-Ru2** can be found in the Supplementary Information). As shown in Fig. 1, the Ru^II^-polypyridyl complexes contain either ethyl ester (**Ru1**) or tertiary butyl (**Ru2**) substituents on their bipyridine ligands^35^. The photosensitizers were chosen to explore the effect of ligand substitution on the charge injection and recombination dynamics. In order to avoid the mutual interaction of two photoexcited Ru-complexes, the **PPV-Ru** systems investigated contained low loadings of Ru^II^ complexes. The **PPV-Ru** systems contained 7.6% of either **Ru1** or **Ru2** related to the overall polymer backbone phenyl units. This loading corresponds to approximately one Ru-chromophore per chromophoric unit as the effective conjugation length of **PPV** is approximately 10 monomer units^36–38^.

### Absorption and emission properties

**Figure 2:**
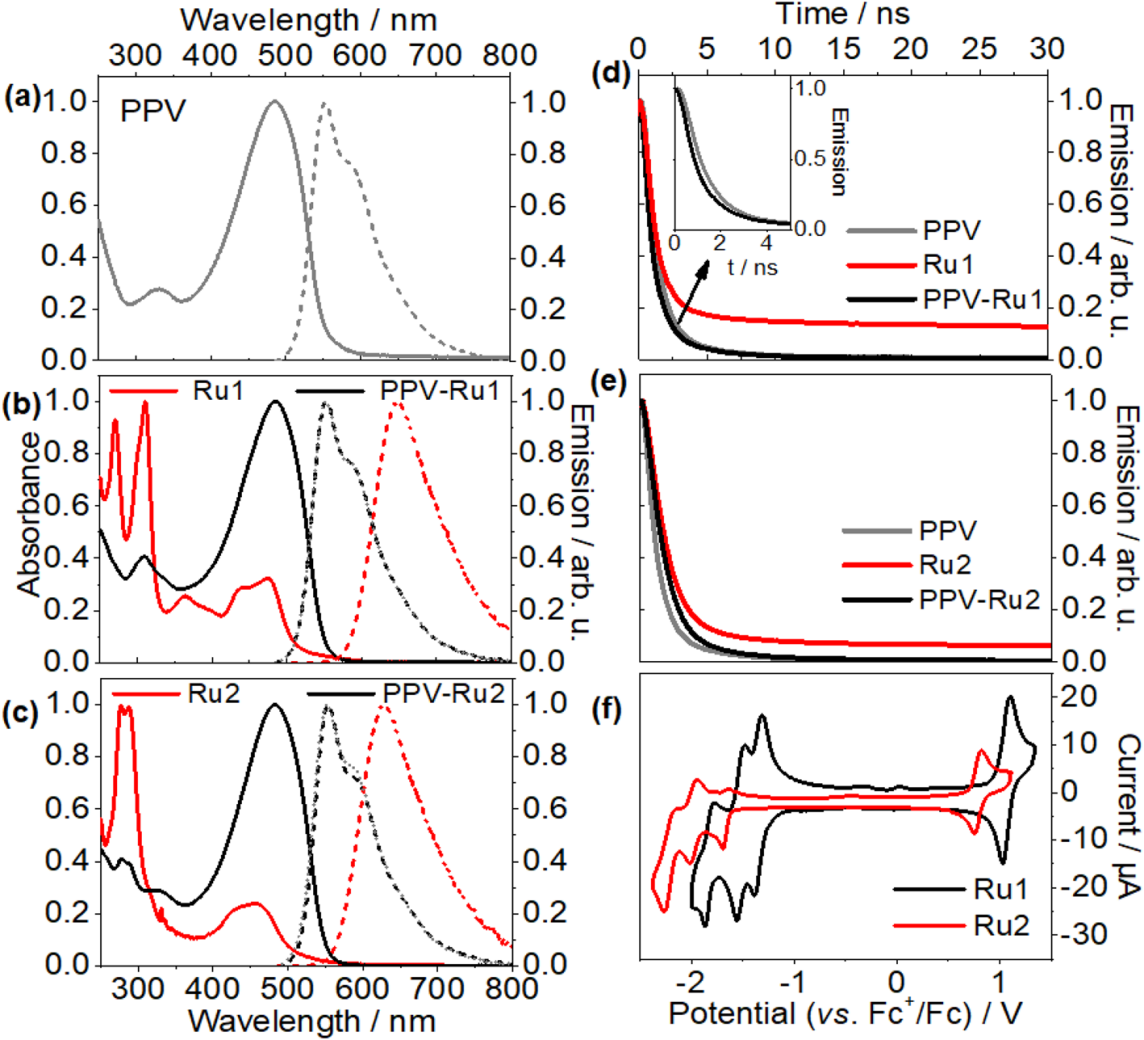
Normalized UV/Vis absorption and emission spectra of (**a**) **PPV**, (**b**) **Ru1**, **PPV**, and **PPV-Ru1**, and (**c**) **Ru2**, **PPV**, and **PPV-Ru2** in CHCl_3_. All emission spectra were obtained upon excitation at 480 nm. Emission decay of (**d**) **Ru1** and **PPV-Ru1**, and (**e**) **Ru2** and **PPV-Ru2** in CHCl_3_ upon excitation at 390 nm. The emission decay was measured using time-correlated single photon counting. The initial fast decay (< 1 ns) arises from the instrumental response function. (**f**) Cyclic voltammogram of 0.25 mM **Ru1** and **Ru2** in acetonitrile using 0.1 M TBABF_4_ as supporting electrolyte.

UV/vis absorption and emission spectra yield first insights into the electronic structure of **PPV-Ru** (see Fig. 2a–c, and for summary see Table 1). The absorption spectrum of **PPV** shows an intense π–π* band at 485 nm. The S_1_ → S_0_ emission reveals a Stokes shift of 311 meV^31–34^ and exhibits vibronic peaks at 552 and 586 nm^39–42^. The emission quantum yield and lifetime were determined to be 0.43 ns and less than 1 ns, respectively. These values are in good agreement with previous literature reports^39–43^; The quantum yield is in good agreement with literature values for emission quantum yields in **PPV** and related systems, which fall in the range between 0.40 and 0.80^39–42^. The short emission lifetime is consistent with observations on, e.g., **MEH-PPV** and dialkoxy-substituted oligo(phenylenevinylene)^43^.

**Table 1:** Photophysical and electrochemical properties of **PPV**, **Ru1** and **Ru2**.

Structure	λ_abs_	λ_em_ (Φ_PL_)^a^	τ_*em*_^b^	*E*_0–0_^c^	*E*_ox_	*E*_red1_	*E*_red2_	*E*_red3_	Δ*G*_inj,e_^d^	Δ*G*_inj,h_^e^
PPV	485	553, 587* (0.43)	< 1	2.30	+ 0.73	− 1.67	–	–	–	–
Ru1	438, 476	646 (0.06)	150	2.17	+ 1.10	− 1.33	− 1.50	− 1.79	0.60^i^; 0.47^ii^; − 1.70^iii^	− 0.11^i^; − 0.24^ii^; − 2.41 ^iii^
Ru2	430, 458	627 (0.02)	139	2.24	+ 0.82	− 1.65	− 1.96	− 2.2	0.25^i^; 0.19^ii^; − 2.26^iii^	0.14^i^; 0.08^ii^; − 2.16^iii^

In nm. UV/vis absorption and emission measurements were measured in CHCl_3_. In V vs. Fc^+^/Fc. Cyclic voltammetry was carried out at a scan rate of 100 mV·s^−1^ and of 0.5 mM complex solutions in acetonitrile containing TBABF_4_ as supporting electrolyte.

^a^Quantum yield (Φ_PL_) of **PPV** was determined using Fluorescein (0.1 M NaOH, Φ_PL_ = 0.95) as reference^46,47^, while the quantum yields of **Ru1** and **Ru2** were determined using [Ru(bpy)_3_]^2+^ (in acetonitrile, Φ_PL_ = 0.03) as reference^23^.

^b^In ns. Emission lifetime was measured using time-correlated single photon counting.

^c^E_0–0_ (in V) is calculated from the crossing point of normalized absorption and emission spectra^48^.

^d^In eV. If only respective Ru^II^ complex is excited: ΔG_inj,e_ = e[E(Ru^+/*^) − E(PPV^0/−^)], where E(Ru^+/*^) = E(Ru^+/0^) − E_0–0_; if only PPV is excited: ΔG_inj,e_ = e[E(Ru^+/0^) − E(PPV^*/−^)], where E(PPV^*/−^) = E(PPV^0/−^) + E_0–0_; and if both PPV and Ru are excited: ΔG_inj,e_ = e[E(Ru^+/*^) − E(PPV^*/−^)].

^e^In eV. If only Ru is excited: ΔG_inj,h_ = e[E(PPV^+/0^) − E(Ru^*/−^)], where E(Ru^*/−^) = E(Ru^0/−^) + E_0–0_; if only PPV is excited: ΔG_inj,h_ = e[E(PPV^+/*^) − E(Ru^0/−^)], where E(PPV^+/*^) = E(PPV^+/0^) − E_0–0_; and if both PPV and Ru are excited: ΔG_inj,h_ = e[E(PPV^+/*^) − E(Ru^*/−^)].

The UV/vis absorption spectra of **Ru1** and **Ru2** exhibit the characteristic features of Ru^II^L_3_ chromophores^7–9,14,15,44^. The intense absorption around 290 nm stems from ligand centered π–π* transitions localized on the bipyridine (bpy) fragments. In the visible range, **Ru1** shows absorption peaks at 430 and 475 nm due to the absorption of metal-to-ligand charge transfer (MLCT) states. Replacing the electron-withdrawing ethyl ester (–COOEt) by electron donating *tert*-butyl groups blue-shifts the MLCT absorption by 100 meV (18 nm). This electronic effect of the substituent is also reflected in the more negative reduction potential of **Ru2** compared with **Ru1** (see electrochemical data below). Both photosensitizers show a weak emission with quantum yields of 0.06 (**Ru1**) and 0.02 (**Ru2**), which is in good agreement with the quantum yield of 0.04 observed for Ru(bpy)_2_(5-CC-phen)](PF_6_)_2_^44^.

The emission lifetime of both complexes exceeds 100 ns and is much longer than the emission lifetime of pristine **PPV** (see Table 1). Upon “sensitization” of **PPV** with the complexes, the UV/vis absorption and emission spectra remain similar to those of the **PPV** backbone, as the comparably weak MLCT absorption band of **Ru1** and **Ru2** overlaps with the intense π–π* transition of **PPV**. The long-lived emission component of the photosensitizer (see Fig. 2d,e) is neither observed for **PPV-Ru1** nor **PPV-Ru2**, which might indicate quenching of the photosensitizers’ long-lived emission. Literature on semiconductor-based dye-sensitized photocathodes generally associates such quenching of photosensitizer luminescence with hole transfer^9,45^. Thus, the apparent absence of any long-lived emission in the sensitized **PPV** polymers is an indication for charge separation within the polymer.

### Electrochemical characterization

Electrochemical reduction/oxidation of the components allows for an estimation of the driving force for different charge transfer pathways in **PPV-Ru** (Fig. 2f). Due to the overlapping absorption spectra of **PPV** and the complexes, it is necessary to consider three possible scenarios: (i) excitation of only the Ru complexes, (ii) excitation of only the **PPV** backbone, and (iii) simultaneous excitation of a chromophoric unit of the **PPV** and a close-by photosensitizer. The estimation of the resultant driving forces for charge injection from the Ru sensitizer into the PPV based on the Gibbs free energies (ΔG) are summarized in Table 1. The Gibbs free energy for electron injection in scenarios (i) and (ii) is positive for both polymers, ΔG_inj,e_ > 0. For **PPV-Ru1** the Gibbs free energy for hole injection ΔG_inj,h_ is negative (albeit small in magnitude), indicating that hole injection is thermodynamically possible. However, ΔG_inj,h_ > 0 in **PPV-Ru2**. Nonetheless, considering the broad oxidation peak of **PPV** (Supplementary Fig. S5a), a slight negative driving force is within the expected error of the calculation, and hence, the estimated ΔG_inj,h_ for **PPV-Ru2,** which is close to zero, does not rule out the possibility that hole injection occurs with very little driving force. Furthermore, cathodic photocurrents for both **PPV-Ru1** and **PPV-Ru2** were observed in the photoelectrochemical experiments which indicates that hole injection does occur in both materials (vide infra). For scenario (iii), simultaneous excitation of Ru^II^ complexes and **PPV**, both hole and electron injection are exergonic with high injection driving forces compared to the aforementioned scenarios. Nonetheless, scenario (iii) is rather unlikely as the photon fluxes used in the presented experiments, i.e. 3.0 × 10^15^ photons cm^−2^ pulse^−1^, are only moderate. Therefore, analysis of the experimental data will consider only scenarios (i) and (ii).

### Transient absorption studies of PPV-Ru in solution

**Figure 3:**
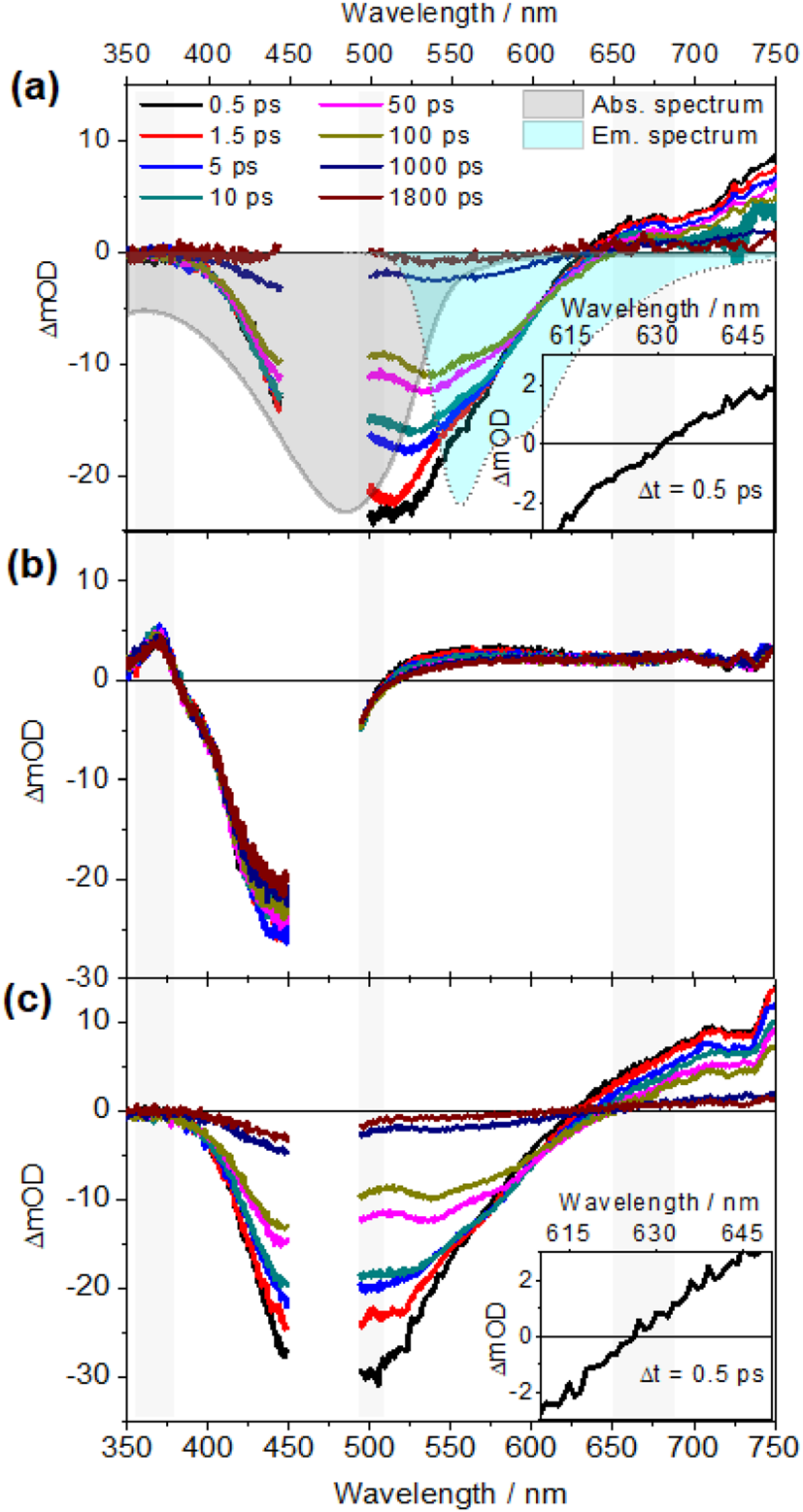
Fs-transient absorption spectra of (**a**) **PPV**, (**b**) **Ru1** and (**c**) **PPV-Ru1** at different delay times measured in CHCl_3_ solution with an excitation wavelength of 480 nm. The shaded areas at 360, 500 and 670 nm indicate the different spectral signatures among **PPV**, **Ru1**, and **PPV-Ru1**. The insets in figures (**a**) and (**c**) show the zero-crossing (ΔOD = 0) at 0.5 ps for **PPV** and **PPV-Ru1**, respectively. TBABF_4_ as supporting electrolyte.

The light-induced charge transfer dynamics of **PPV-Ru** dissolved in CHCl_3_ were investigated by transient absorption spectroscopy upon excitation at 480 nm. First, transient absorption data were recorded for each individual component, **PPV** and each of the photosensitizers, (and this is further discussed in the Supplementary Information, see Supplementary Figs. S11–S15). Briefly, the transient absorption spectra of **PPV** measured in CHCl_3_ show an initial ground-state bleach (GSB), stimulated emission (SE) at 530 nm (peak) as well as 565 nm (shoulder), and an excited-state absorption (ESA) band beyond 600 nm (Fig. 3a). Quantitative analysis of the data was performed by globally fitting the data to a three-exponential decay (Table 2). This approach yields the characteristic time constants τ_1_ = 3.8 ps, τ_2_ = 72 ps, and τ_3_ = 673 ps. The corresponding decay-associated spectra are shown in Supplementary Fig. S11c. The pump-probe data of **PPV** is consistent with literature reports^26–28,36,37^: The early relaxation originates from a strong coupling between electronic and vibrational states. Hence, the fast kinetic processes are attributed to delocalized exciton states (self-trapping, ~ 100 fs) and vibrational cooling (few ps)^49^. Subsequently, the excited state dynamics is dominated by either interchain or intrachain energy transfer on a characteristic timescale of tens of ps^50,51^. This electronic energy transfer occurs prior to emission which generally stems from localized low-energy sites^52,53^.

**Table 2:** Characteristic time constants (τ) of **PPV**, **Ru1** and **Ru2** extracted from global fit with a sum of exponential functions of TA spectra measured in solution.

Structure	*τ*_1_/ps	*τ*_2_/ps	*τ*_3_/ps
PPV	3.8	72	673
Ru1	0.2	8	Infinite
Ru2	0.2	78	Infinite

The transient absorption spectra of both **Ru1** (Fig. 3b) and **Ru2** (Supplementary Fig. S13) exhibit a broad and rather unstructured ESA beyond 515 nm due to ligand-to-metal charge transfer transitions and a GSB centered at around 450 nm. The ESA band at 370 nm is assigned to the π_1_* → π_2_* transition at the bpy^•−^ fragment^9,44,46,47^. The minor transient absorption signal decay within the experimentally accessible time window of 1.8 ns reveals the presence of a long-lived ^3^MLCT state typical for Ru tris-diimine complexes^9,44^. Quantitative analysis by global fitting with two-exponential decay functions and an infinite component (reflecting the presence of the long-lived state, see Table 2) results in characteristic time constants (τ_1_ = 0.2 ps, τ_2_ = 8 ps for **Ru1**, and τ_1_ = 0.2 ps, τ_2_ = 78 ps for **Ru2**). The corresponding decay-associated spectra are shown in Supplementary Figs. S12 and S13. The pump-probe data of the photosensitizers is consistent with literature reports on similar Ru^II^ complexes^9,46,47,54–56^: The fast process (τ_1_) reflects contributions from intersystem crossing (ISC, ^1^MLCT → ^3^MLCT) and intramolecular vibrational redistribution. The second process (τ_2_) corresponds to thermal relaxation within the ^3^MLCT manifold and leads to the population of the thermally equilibrated ^3^MLCT state.

Having summarized the photoinduced kinetics recorded for the individual molecular fragments, we turn our discussion to the combined system **PPV-Ru**. The transient absorption spectra of **PPV-Ru1** (Fig. 3c) and **PPV-Ru2** (Supplementary Fig. S15) are dominated by the spectral signature of **PPV** since the loading of the polymer with either of the photosensitizers is comparably low. Nonetheless, slight differences in the transient absorption spectra of **PPV-Ru** are observed compared to the reference **PPV** data; In particular the spectral shape at the red flank of the negative differential absorption band differs slightly due to spectral overlap of GSB contribution from **PPV** and contributions from photosensitizer-associated excited-state absorption. The zero-crossing (ΔOD = 0) of **PPV-Ru** thus shifts to a shorter wavelength at early delay time (Δt = 0.5 ps) compared with **PPV**, i.e., from 631 to 623 nm for **PPV-Ru1**.

The background contribution from **PPV** in the transient absorption spectra of the **PPV-Ru** systems makes the light-induced charge transfer processes difficult to analyze. To overcome this, the data obtained for **PPV** was subtracted from the transient absorption signal of **PPV-Ru**. Prior to subtraction the data is normalized to its early time (Δt = 0.5 ps) signal amplitude at 515 nm. At this probe wavelength there is no contribution of the transient absorption signal from the respective **Ru**^II^ complex (see Fig. 3b). The subtracted, i.e. differential transient absorption spectra (ΔΔOD = ΔOD_[PPV-Ru]_ − ΔOD_[PPV])_, are determined as follows:

1 $$\Delta \Delta OD\left( {\Delta t,\lambda } \right) = \frac{{\Delta OD\left( {\Delta t,\lambda } \right)_{{\left[ {PPV - Ru} \right]}} }}{{\Delta OD\left( {\Delta t = 0.5\,{\text{ps}},\lambda = 515\,{\text{nm}}} \right)_{{\left[ {PPV - Ru} \right]}} }} - \frac{{\Delta OD\left( {\Delta t,\lambda } \right)_{{\left[ {PPV} \right]}} }}{{\Delta OD\left( {\Delta t = 0.5\,{\text{ps}},\lambda = 515\,{\text{nm}}} \right)_{{\left[ {PPV} \right]}} }}.$$

**Figure 4:**
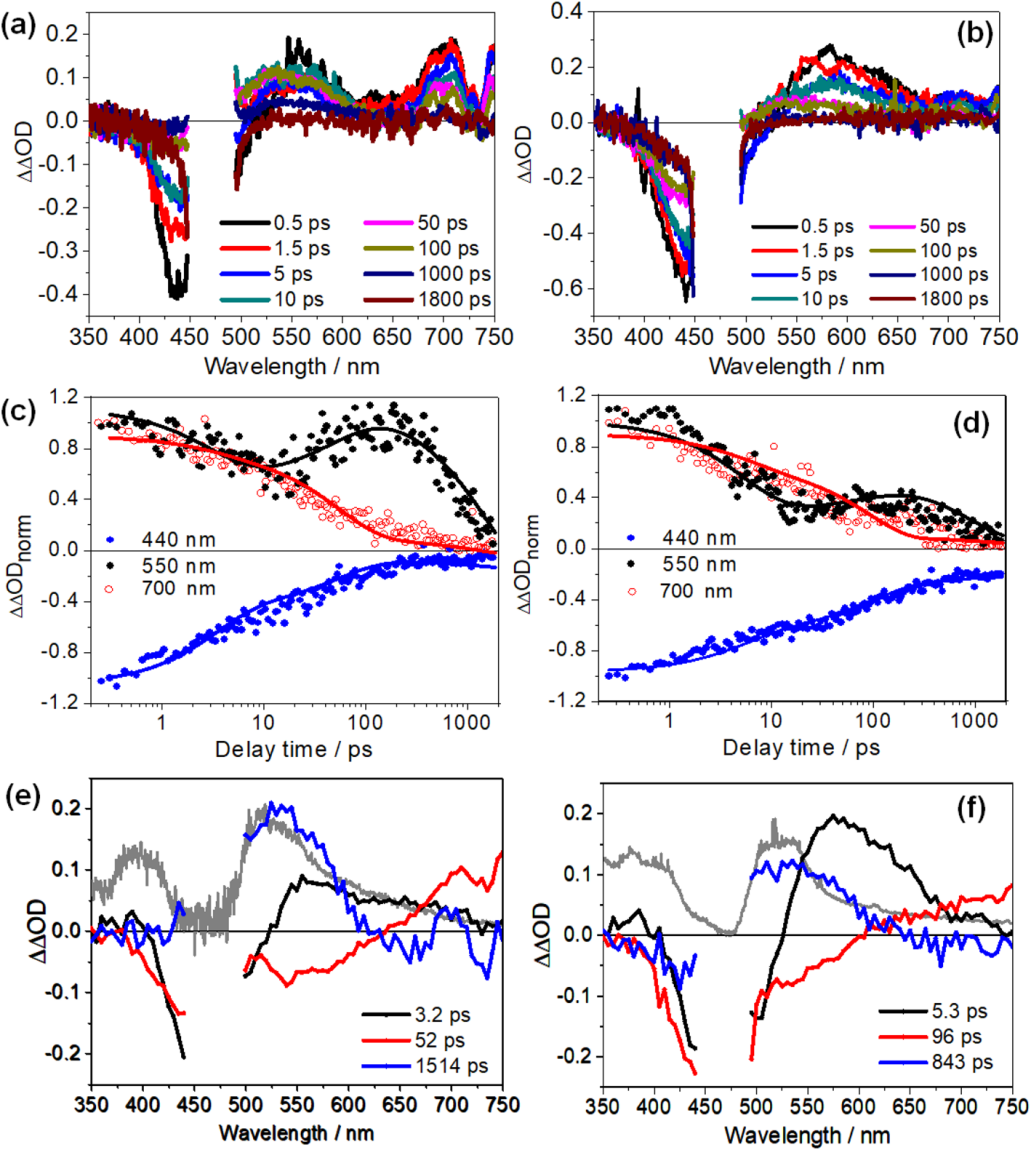
Differential fs-transient absorption spectra of (**a**) **PPV-Ru1** and (**b**) **PPV-Ru2** at different delay times. Normalized kinetic traces at different probe wavelength and decay-associated spectra (DAS) resulting from the global fit with three time constants for (**c**,**e**) **PPV-Ru1** and (**d**,**f**) **PPV-Ru2**. The resulting time constants in the DAS are statistically taken from three independent measurements. The pump pulses are centred at 480 nm. The grey spectra in panel (**e**) and (**f**) depict the differential absorption spectra of **Ru1** and **Ru2**, respectively, held at a potential of slightly more negative than that of the first reduction potential (Supplementary Figs. S7 and S8), which is arbitrarily scaled to fit the transient absorption data.

From the ΔΔOD signal the excited state interactions between the photosensitizer and the **PPV** in the photoexcited **PPV-Ru** system can be deduced (see Fig. 4) irrespective of whether the initial excitation occurs on the PPV backbone, or in the pendant photosensitizer. The ΔOD spectra recorded as a function of Δt were globally analysed using a tri-exponential fit. The decay-associated spectra (DAS) generated from the fit are shown in Fig. 4e,f. In contrast to the dynamics of **Ru1** and **Ru2** in solution, the ΔOD exhibits a rapid quenching of the negative signal contribution associated with GSB at 450 nm, which occurs jointly with a decay of the positive ΔΔOD at 560 and 700 nm. However, from 10 ps up to 100 ps, the ΔΔOD signal at 550 nm increases again, while it continues to decrease in other spectral regions. This increase of ΔΔOD(550 nm) on a sub-100 ps timescale is followed by a slow decay of the overall signal.

The first kinetic process characterized by τ_1_, 3.3 ± 1.2 ps, for **PPV-Ru1** and 4.0 ± 1.3 ps for **PPV-Ru2**, is assigned to energy dissipation following photoexcitation. The second process is characterized by a decay of the negative ΔΔOD at around 450 nm and the built-up of positive ΔΔOD observed at 550 nm, which is reflected in the kinetic traces in Fig. 4c,d. This signal rise at 550 nm appears more prominent for **Ru1**, but is also observed for **Ru2**, and is accompanied by a decay of the positive ΔΔOD at wavelengths longer than 610 nm. The positive ΔΔOD signal, which builds up with τ_2_, correlates well with the spectral change upon electrochemical reduction of either **Ru1** or **Ru2** (grey spectra in Fig. 4e,f). Thus, we conclude that τ_2_ is the characteristic time constant for formation of the bpy radical anion (bpy^•−^), i.e. τ_2_ reflects hole injection to the **PPV** backbone in the photoexcited **PPV-Ru** polymers. Hole injection generates a CSS with the hole residing in the **PPV** and a reduced Ru-complex. The slowest process (τ_3_) is mainly characterized by a decay of positive ΔΔOD centered at 525 nm indicating the decay of the signal produced by the reduced ligand and hence the decay of the CSS. τ_3_ reflects the lifetime of the CSS and amounts to 994 ± 203 ps for **PPV-Ru2** and 1584 ± 201 ps for **PPV-Ru1**. This lifetime is comparable to the reported CSS lifetime in conventional Ru polypyridyl complex-sensitized p-type semiconductors for photocatalytic hydrogen evolution^9,46,47,54–56^. For example, in NiO photocathodes sensitized with [Ru(bpy)_3_]^2+^-based complexes, the CSS h^+^(NiO)/bpy^•−^ is characterized by lifetimes spanning from hundreds of ps to a few ns depending on the bpy ligand substitution pattern (vide infra)^45,55–57^.

The respective spectral changes reflecting the decay of the CSS do not involve the positive band below 425 nm, which is observed upon electrochemical reduction of the Ru chromophores in solution (compare the blue and grey spectra plotted in Fig. 4e,f). The absence of this feature might be partially rationalized considering the strong bleach induced by the oxidation of **PPV** between 405 and 540 nm (Supplementary Fig. S5). Superposition of the this bleach with the absorption of the reduced Ru chromophores is also responsible for the slight shift between the spectral maxima of the τ_3_-component (blue line) and the spectrum of the reduced chromophore (grey line in Fig. 4e,f).

In principle, energy transfer from a photoexcited Ru complex to the **PPV** backbone to form a PPV triplet state could be a deactivation pathway working in parallel with hole injection. Similar processes have been observed in π-conjugated polymers functionalized with photosensitizers, e.g., for applications in photovoltaic and electroluminescent devices^58–62^. Thus, triplet energy transfer (after ultrafast intersystem crossing) from the photoexcited Ru sensitizers to the conjugated polymer must be considered here as well. A key observation towards increased PPV-triplet yields in **PPV-Ru** vs. **PPV** should arise from ns transient absorption^63,64^. However, transient absorption data recorded in the ns to µs time range revealed no difference between **PPV** and **PPV-Ru1** that would point to formation of **PPV** triplet states (Supplementary Fig. S17). Hence, we conclude that the observed spectral changes reflected in the ΔΔOD spectra do not stem from long-lived **PPV** triplets, which are sensitized by the Ru chromophores, but rather from hole injection to the polymer.

Hole injection in **PPV-Ru1** (τ_2_ = 48 ± 4 ps) is slightly faster than in **PPV-Ru2** (τ_2_ = 86 ± 9 ps), while the CSS recombines somewhat faster in **PPV-Ru2** (994 ± 203 ps) than in **PPV-Ru1** (1587 ± 201 ps). The faster hole injection rate in **PPV-Ru1** can be associated with the higher driving force for hole injection indicated by the more negative Gibbs free energy (see Table 1) as compared to **PPV-Ru2**. In addition, variations of the ligand substitution are known to alter hole injection rates in NiO-based photocathodes sensitized with [Ru(bpy)_3_]^2+^-derived chromophores^45,55–57^: e.g., [Ru(bpy)_2_(dcb)]^2+^ (dcb = 4,4′-dicarboxy-2,2′-bipyridine)-sensitized NiO does not show hole injection when anchored via the dcb ligand to the NiO surface. However, hole injection is observed for [Ru(dcb)_3_]^2+^ as a sensitizer^44^. A related report by Dempsey and colleagues showed hole injection to NiO using [Ru(flpy)_2_(dcb)]^2+^ (flpy = 4,4′-bis(trifluoromethyl)-2,2′-bipyridine, dcb = 4,4′-dicarboxy-2,2′-bipyridine) as sensitizer, but not for [Ru(bpy)_2_(dcb)]^2+^. The authors associated their result with the stronger electron withdrawing character of the utilized –CF_3_ on bpy-ancillary ligands^56^. Considering sensitized metal-oxide photocathodes such effects of ligand-substitution and related findings associated with the lifetime of the CSS^4^ can be rationalized by taking into account a fixed orientation of the photosensitizer (and hence the ligand-centered radical anion) with respect to the semiconductor. Such arguments are not straightforward in **PPV-Ru**, as hole transfer will occur through space and it is not a priori evident if the closest distance between the photosensitizer and a chromophoric unit of the (generally) coiled **PPV** is via the clicked phenanthroline or the bipyridine ligands. Nonetheless, the experimental data provides evidence for sensitive structural control of the charge injection dynamics via ligand-design when considering hole injection, not only to NiO but also into potential soft-matter electrodes.

### Transient absorption studies of PPV-Ru in films

**Figure 5:**
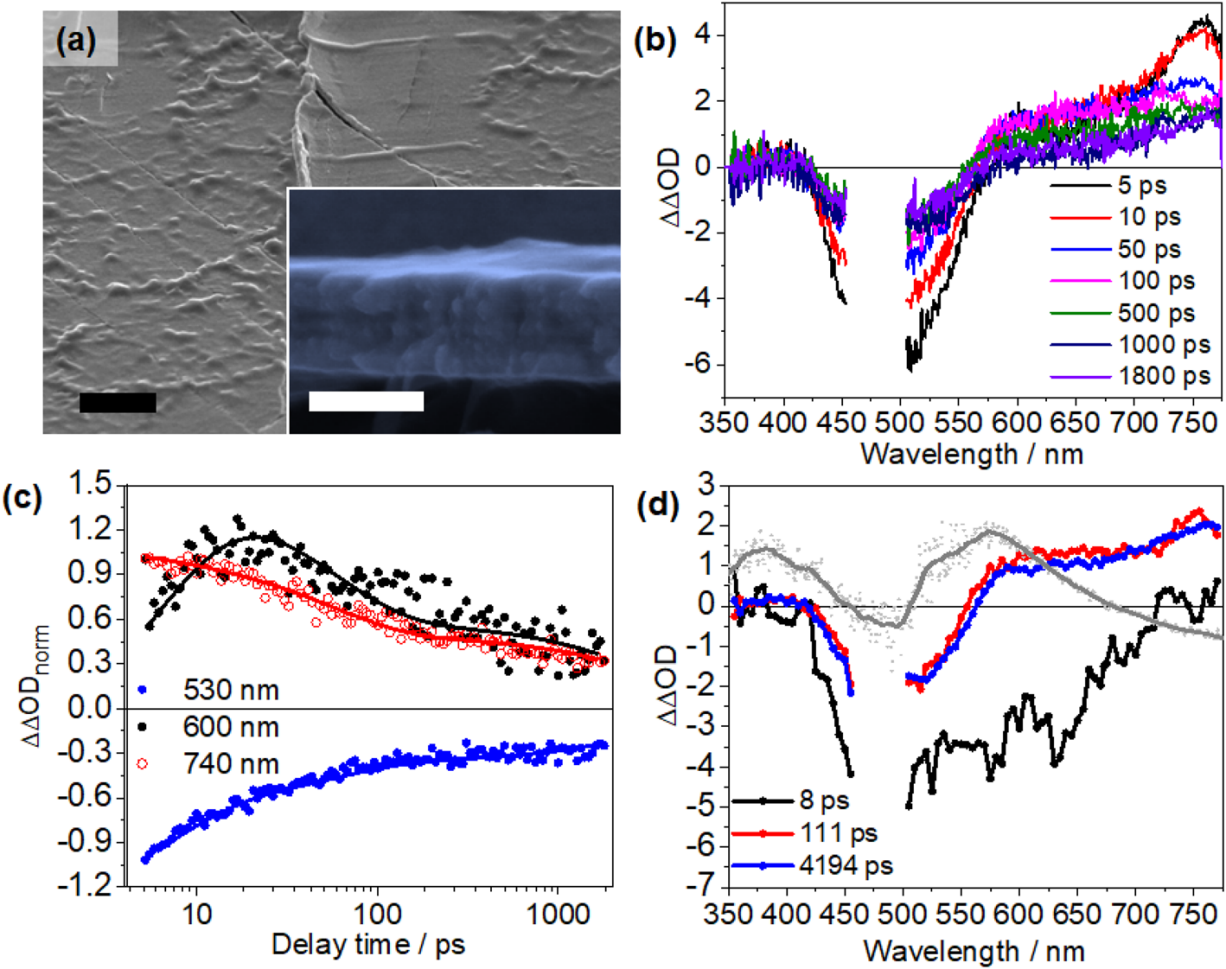
(**a**) Micromorphology of a **PPV-Ru1** film dropcasted onto PET/ITO substrate. The inset depicts the cross-section image of **PPV-Ru1** photocathodes. The black and white scale bar in panel (**a**) indicate 3 µm and 300 nm, respectively. (**b**) Fs-transient absorption spectra, (**c**) normalized kinetic traces recorded at different probe wavelength, and (**d**) decay-associated spectra (DAS) resulting from the global fit of the transient absorption data using a tri-exponential model. The grey spectra in panel (**d**) depict the absorption-difference spectra upon chemical reduction of **Ru1** (at − 1.4 V vs. Ag|AgCl) in the **PPV-Ru1** film. The absorption-difference spectra (Supplementary Fig. S10) are arbitrarily scaled to the figure.

To evaluate the potential of **PPV-Ru** as materials for all-polymer photoelectrodes, we studied the photoinduced charge-transfer processes in drop-cast films of **PPV-Ru1** (see Fig. 5). Films were prepared by drop-casting a solution of **PPV-Ru1** in CH_3_Cl (10% w/v) onto PET/ITO substrates. This gave relatively smooth films with a thickness of 300 ± 16 nm, which is in the lower range of typical thicknesses for NiO-based photocathodes^9,10,45^. Following the same data analysis as laid out above, ΔΔOD spectra are obtained for **PPV-Ru1** (Fig. 5b). The ΔΔOD shows negative features between 400 and 580 nm, which are broader than for **PPV-Ru1** in solution. Furthermore, the ΔΔOD kinetics reveal differences between **PPV-Ru1** in film and in solution (Fig. 5c). For **PPV-Ru1** films, the ΔΔOD features a rapid initial decay of the ground state bleach at 530 nm and a positive ΔΔOD band at 740 nm. The ΔΔOD signal at 600 nm increases on a timescale of up to 20 ps and is followed by an overall decay of the signal.

The ΔΔOD data of **PPV-Ru1** is analysed using a tri-exponential fit (see Fig. 5d). The first decay component characterized by τ_1_ = 8.3 ± 2.6 ps reflects a build-up of positive ΔΔOD at around 600 nm accompanied by a loss in ground-state bleach. The signal build-up at 600 nm can be correlated with the spectral changes upon electrochemical reduction of **Ru1** in **PPV-Ru1** (grey spectrum in Fig. 5d). However, the positive DAS(τ_2,3_)-feature observed above 600 nm extends further to the blue than the signal reflecting the reduction of the **Ru**-chromophore in **PPV-Ru1**. This red shift could be due to the contribution of a broader GSB feature of Ru1 measured in film which 410 and 575 nm (see Supplementary Fig. S19). Therefore, τ_1_ is assigned to hole injection. The subsequent processes associated with τ_2_ and τ_3_ are characterized by a decay of the reduced **Ru1**, i.e. the positive ΔΔOD band > 550 nm. Charge recombination, i.e. full decay of the CSSs due to recombination occurs bi-exponentially with τ_2_ = 107 ± 19 ps and the significantly longer τ_3_ = 4.8 ± 1.1 ns. Hence, it is apparent that the rate of hole injection from the photosensitizer to the polymer backbone and (partial) charge recombination in **PPV-Ru1** are faster in the film than in solution.

We propose that the difference in the hole-injection rates for the solution phase and drop-caste polymer can be rationalized by considering “frozen geometries” in the polymer film; In these ‘geometries’ a sensitizer and a chromophoric unit of the **PPV** are arranged favourably for electronic coupling. As a result, hole injection and charge recombination occur more rapidly in the drop-caste polymer than for the polymer in solution. In solution the polymer can fluctuate in and out of such “optimal” geometries. These slow structural fluctuations available to the polymer in solution allow the system to sample a large conformational space, which ultimately leads to charge recombination with a characteristic time-constant of roughly 1 ns. In the **PPV-Ru1** films sample, the hole injected into the **PPV** backbone upon creation of the CSS initially resides close to the Ru^II^ complex. It can then be transferred to other chromophoric units of the polymer which were not involved in the initial hole injection process. Upon such hole migration^65,66^, the hole residing in the **PPV** becomes spatially separated from the reduced Ru chromophore and hence charge recombination becomes hindered. Depending on the actual substitution pattern of **PPV** hole migration occurs as fast as within 100 ps^65,66^. For BEH-PPV, i.e. poly[2,5-bis(2′-ethyl-hexyl)-1,4-phenylenevinylene, a substitution pattern similar to the one used here, hole characteristic migration times of 180 ps have been determined in a pristine thin film^67,68^. Thus, hole migration occurs on a time-scale comparable to the decay of the primary CSS in **PPV-Ru1** films. The presence of two distinct charge recombination channels in **PPV-Ru1** films is thus attributed to the presence of two different ensembles of CSS in the polymer: one with optimal electronic coupling between the hole and the reduced chromophore (responsible for the comparably fast decay characterized by τ_2_), and another set of geometries in which the hole has been further separated from the reduced Ru^II^ photosensitizer by hole transfer in the polymer (responsible for the slow charge recombination characterized by τ_3_).

### Photoelectrochemical measurements

**Figure 6:**
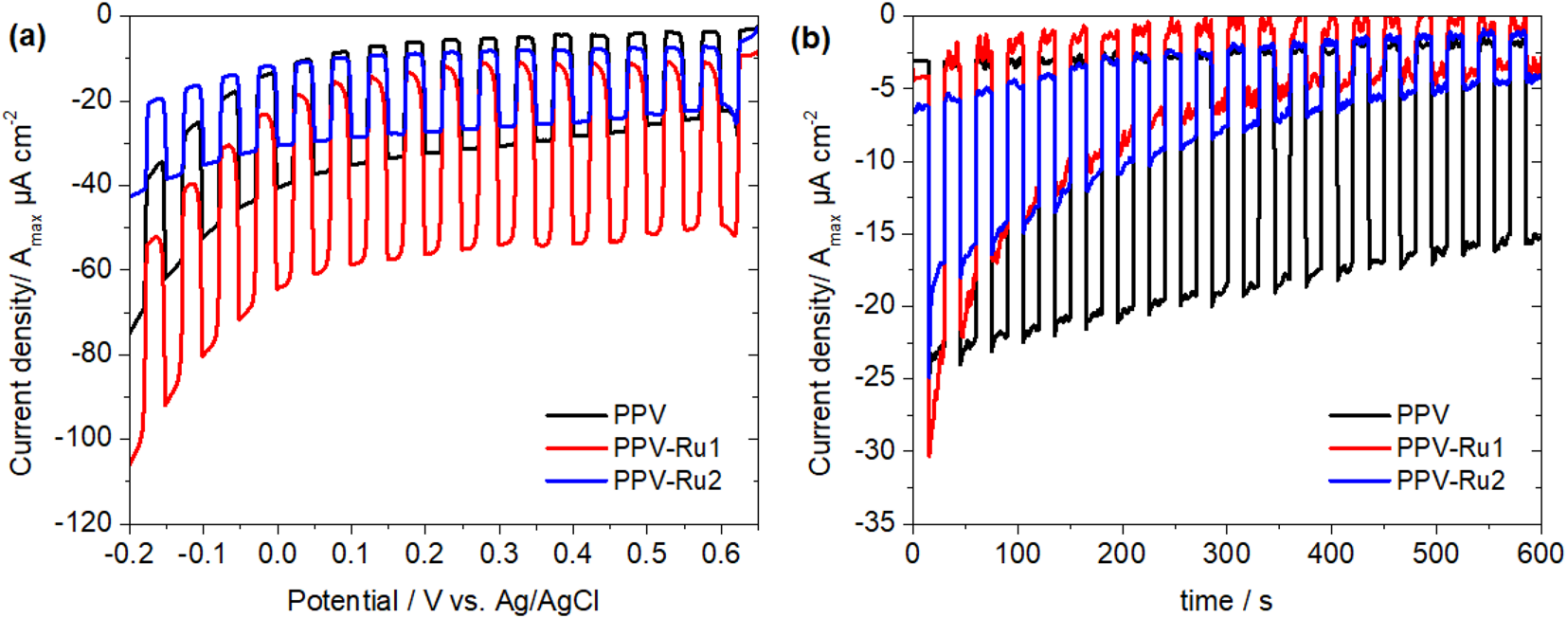
Results of the photoelectrochemical measurements performed on drop-casted films of **PPV**, **PPV-Ru1** and **PPV-Ru2** in Co^III^/Co^II^ electrolyte under chopped illumination (1000 nm > λ > 300 nm, 1000 W⋅m^−2^). (**a**) Linear scan voltammogram (scan speed 20 mV/s) and (**b**) chronoamperometric measurement at 0.5 V vs. Ag/AgCl applied potential. The obtained currents were divided by the film surface area to obtain current densities, and further divided by the maximum absorbance (A_max_) of the films to account for film thickness. Shown are the average values of four individual measurements.

The charge-transfer behavior of the photoexcited polymers was investigated using linear scan voltammetry (LSV) and chronoamperometry under chopped irradiation (white light, 1000 W m^−2^). The experiments were carried out on drop-caste films of the polymers using a three-electrode spectroelectrochemical cell and a Co^III^/Co^II^ redox couple as a sacrificial electron acceptor (Fig. 6).

In the LSV experiments, all polymers show a cathodic photocurrent that is independent of the applied potential, while the dark current increases steeply below ca. 0 V vs. Ag/AgCl. Of the three polymers, **PPV-Ru1** shows the highest photocurrent with 42 µA cm^−2^A_max_^−1^ (6.5 µA cm^−2^ current density before normalization to the absorbance, see Supplementary Fig. S22), which is in line with the faster hole injection and slower charge recombination observed in the TA experiments. Compared to the conventional [Ru(bpy)_3_]^2+^-sensitized NiO photocathode, the resulting current density in **PPV-Ru1** is at this stage of the proof-of-concept study notably lower (6.5 vs 120 µA cm^−2^)^9^. Nonetheless, this current density is found on par to the organic push–pull dye-sensitized NiO photocathodes which yield photocurrent density in the range of 2.2–6 µA cm^−2^^69,70^.

In the chronoamperometric experiments, **PPV-Ru1** also initially shows the highest photocurrent density. However, this decreases by 90% during the duration of the experiment (10 min). While **PPV-Ru2** shows a similar behavior, the non-functionalized **PPV** film shows a much slower decrease in photocurrent density (35% loss after 10 min). The decrease in photo-current density during the chopped-light measurements indicates that the investigated polymers are rather unstable under irradiation, which is supported by the strong decrease in absorbance of the PPV absorption band in post-operando UV–Vis spectra of the films (see Supplementary Fig. S23). Therefore, this instability is likely due to the poor photostability of the PPV backbone itself^67,68^. We ascribe the acceleration of the decay in **PPV-Ru1** to the prolonged charge-separated state lifetime in comparison with pristine **PPV**, which could lead to faster degradation of the **PPV** backbone in it’s oxidized state. The extension of the charge-separated state lifetime is therefore a double-edged sword, leading to both an increase in activity and a decrease in stability. However, because of the modular nature of these soft matter materials, in future work both the polymer backbone and sensitizer components can be synthetically optimized to produce more stable soft-matter photocathodes.

## Conclusions

We have demonstrated the photosensitization of **PPV** with different polypyridyl Ru^II^ complexes through CLICK chemistry. The charge transfer dynamics in the polymer were unravelled using time-resolved optical spectroscopy. In both polymers a CSS was formed by light-driven hole injection from the photosensitizer into the **PPV** backbone. While photoinduced hole transfer in drop-cast films of **PPV-Ru1** took place in less than 10 ps, it was found to take place on a sub-100 ps timescale for samples in solution. The CSS lifetimes in solution and in drop-caste films were comparable to that observed in very recent studies^45,55–57^ for conventional NiO-based photocathodes. Altering the ligands of the Ru^II^ complexes modified hole injection and recombination rates within the sensitized polymers. An electron-withdrawing group on the bipyridyl ligand gave rise to faster hole injection into the polymer backbone and slower charge recombination. As a proof-of-concept, we showed that the **PPV-Ru** system works as a functional photocathode indicated by the generation of a cathodic current in a photoelectrochemical experiment. Thus, we have shown that light induced charge transfer occurs between PPV and ruthenium complexes attached to the polymer via side chains. This leads to sufficiently long-lived charge separated states to allow electron transfer to acceptor species in solution. Furthermore, we have demonstrated a modular design approach implementing CLICK chemistry at innocent phenanthroline ligands to attach two different ruthenium complexes (**Ru1** and **Ru2** with chemically very different ligand substitution patterns). This indicates that sophisticated supramolecular photocatalyst architectures can be introduced^69^, permitting the implementation of well-established synthetic chemistry for tris-heteroleptic ruthenium building blocks^70^.

We intend to further exploit the concept introduced here to develop “molecularly functionalized” hole-conducting polymer photocathodes as an alternative to conventional NiO photoelectrodes. Ultimately, this research should lead to the realization of soft-matter photoelectrochemical cells for solar fuel generation.

## Experimental section

### Synthesis and preparation of PPV, Ru’s, and PPV-Ru’s

Detail of synthetic procedure of PPV, Ru’s, and PPV-Ru’**s** including materials and instrumentation is described in the Supplementary Information.

### Spectroscopy and (Spectro)electrochemical measurements

#### UV/vis absorption and emission measurement

UV/vis absorption and emission spectra of **PPV, Ru1, Ru2, PPV-Ru1** and **PPV-Ru2** were measured in CHCl_3_ using a Varian-Cary UV–Vis-NIR spectrometer and FLS980 photoluminescence spectrometer (Edinburg Instrument), respectively^71^.

#### Spectroelectrochemical measurements

Each solution contained 0.1 M tetrabutylammonium tetrafluoroborate (Sigma-Aldrich, for electrochemical analysis, ≥ 99.0%) in CH_3_Cl as supporting electrolyte. The setup for both electrochemical and spectroelectrochemical measurements was described before^72,73^: A thin-layer 1 mm path length quartz glass spectroelectrochemical cell (Bioanalytical Systems, Inc.), equipped with a Pt counter electrode, a Ag|AgCl-pseudoreference electrode and a glassy carbon working electrode with a slit of 2 mm × 5 mm, was used. The redox potentials were determined by cyclic voltammetry using a VersaSTAT 3 potentiostat (Princeton Applied Research). UV/Vis spectra were collected during reduction of the complexes, i.e. during chronoamperometry at various potentials. The UV/Vis spectra were obtained using a single channel fiber optic spectrometer (Avantes Inc., AvaSpec-ULS2048XL) with a deuterium-halogen light source (Avantes Inc., AvaLight DH-S-BAL). For UV/vis spectroelectrochemistry of **PPV-Ru1** films, 20 µL of **PPV-Ru1** solution (either 10% or 20% w/v) in CH_3_Cl was drop-caste on transparent PET/ITO (Indium-doped Tin Oxide) substrate. The **PPV-Ru1** film thickness was determined from the cross-section images obtained by using JEOL JSM6300F scanning electron microscope operating at 5.0 kV accelerating voltage. The UV/vis spectra were obtained in transmission mode through the PET/ITO/**PPV-Ru1** working electrode using the same spectrometer and light source as mentioned above.

#### Time-resolved emission measurements

Time-resolved emission measurements were carried out by using time-correlated single-photon counting (TCSPC)^45^. A Ti:Sapphire laser (Tsunami, Newport Spectra-Physics GmbH) was used as the light source. The repetition rate is reduced to 400 kHz by a pulse selector (model 3980, Newport Spectra-Physics GmbH). Afterwards, the fundamental beam of the Ti–Sapphire oscillator is frequency doubled in a second harmonic generator (Newport Spectra-Physics GmbH) to create the 390-nm pump beam. The emission was detected by a Becker and Hickel PMC-100-4 photon-counting module. The emission lifetimes were determined by fitting a monoexponential decay to the data.

#### Transient absorption measurements

The setup for femtosecond (fs)—transient absorption measurements of **PPV, Ru1, Ru2, PPV-Ru1**, and **PPV-Ru2** both in solutions and on films was previously described^74–76^: The 800 nm output of an amplified Ti:Sapphire laser (Legend, Coherent Inc.) was split into two beams, one of which was used to pump an optical-parametric amplifier (TOPAS-C). The TOPAS output pulses were spectrally centered at 480 nm with a pump-pulse energy of typically 1.2 μJ and used as pump pulses in the fs pump–probe experiments. This pump beam was directed over a 600 mm delay line in order to realize the temporal delay of up to ~ 2 ns between the pump and the probe pulses. The residual fraction of the fundamental was employed for supercontinuum generation in a CaF_2_ window used as a broad-band probe in the transient absorption experiments, in which the typical probe intensities fall into the range of hundred nJ. The probe light was then split into two beams, one of which was focused into the sample by means of a 500 mm focal length spherical mirror, while the second beam was used as reference. The mutual polarizations of pump and probe were set to magic angle. Probe and reference intensities were detected on a double-stripe diode array and converted into differential absorption (DA) signals using a commercially available detection system (Pascher Instruments AB, Sweden). Chirp correction and subsequently a global fit routine using a sum of exponentials was carried out for data analysis^75,76^. To avoid prominent contributions from coherent artifacts^77,78^, the pulse overlap region (± 250 fs around time zero) was excluded from the data fitting procedure. For solution measurement, samples were dissolved in CHCl_3_ in a quartz cell with 1 mm optical path length, while the **PPV, Ru1, Ru2, PPV-Ru1**, and **PPV-Ru2** film were prepared by drop casting 100 µL of solution (10% w/v) on ITO coated microscope slide. Both solution and film samples were moved during scans to allow the pump-probe beam to hit the fresh sample area.

The nanosecond (ns) transient measurements were carried out to investigate the long-lived species in **PPV** and **PPV-Ru** in solutions upon photoexcitation. All measurements were undertaken in 1 cm path length fluorescence cuvettes. The setup for ns—transient absorption measurements was previously described^79,80^: The pump pulse was centered at 480 nm and generated by a Continuum Surelite OPO Plus apparatus (pumped by an Nd:YAG laser system, pulse duration 5 ns, repetition rate 10 Hz). A 75 W xenon arc lamp was used as the probe light. Spherical concave mirrors were utilized to focus the probe light into the samples as well as to the monochromator (Acton, Princeton Instruments). The probe light was detected by a Hamamatsu R928 photomultiplier. The signal was amplified and processed by a commercially available detection system (Pascher Instruments AB). For all measurements, the pump pulse energy was kept at 0.4 mJ. A spectral band of 20 nm around the pump wavelength is omitted from the data analysis due to pump-scatter in this spectral range.

#### Photoelectrochemical measurements

Photoelectrochemical measurements were conducted in a three-electrode cell. The working electrode was a polymer-masked (Surlyn) PET/ITO substrate which was coated with the **PPV(-Ru)** polymer films by drop-casting: 100 µL of polymer solution (1 mg/mL) in CHCl_3_ was drop-casted on a masked PET/ITO substrate of ca. 1 × 3 cm size. After evaporation of the solvent the mask was removed to yield substrates of ca. 1 × 1 cm size. The reference electrode was a leakless Ag|AgCl electrode (EDAQ) and the counter electrode was a Pt wire. A Co^III^/Co^II^ redox couple (0.25 M tris(2-(1H-pyrazol-1-yl)pyridine) cobalt(II) di[bis(trifluoromethane)sulfonimide, 0.06 M tris(2-(1H-pyrazol-1-yl)pyridine)cobalt(III) tri[bis(trifluoromethane)sulfonimide), was added to the supporting electrolyte solution (0.1 M LiTFSI, and 0.5 M 4-*tert*-butyl pyridine in dry acetonitrile). Linear scan voltammograms were recorded at 20 mV/s scan speed with chopped light illumination (1000 nm > λ > 300 nm, 1000 W m^−2^, 5 s cycle time) provided by a light source connected to a Zahner potentiostat (Zennium Pro). The chronoamperometric measurements were performed with an applied potential of 0.5 V vs. Ag|AgCl and using the same light source with a cycle time of 30 s.

## Supplementary Information

Supplementary Information.
