# Supplementary material for: Photocathodes beyond NiO: charge transfer dynamics in a π-conjugated polymer functionalized with Ru photosensitizers
Source: Sci Rep. 2021 Feb 2;11:2787. doi: 10.1038/s41598-021-82395-x (PMC7854750; doi:10.1038/s41598-021-82395-x)
Supplement: Supplementary file 1 — Supplementary Information. [file 41598_2021_82395_MOESM1_ESM.pdf]

[Supporting Information]

Photocathodes Beyond NiO – Charge Transfer Dynamics in a π-Conjugated Polymer Functionalized with Ru Photosensitizers

Ruri A. Wahyuono[1,2,3], Bianca Seidler[1,2,4], Sebastian Bold[1,2,5], Andrea Dellith[1], Jan Dellith[1], Johannes Ahner[6,7], Pascal Wintergerst[4], Grace Lowe[4], Martin D. Hager[6,7], Maria Wächtler[1,2], Carsten Streb[4], Ulrich S. Schubert[6,7], Sven Rau[4], Benjamin Dietzek[1,2,7,*]

[1] *Leibniz Institute of Photonic Technology (IPHT) Jena e.V., Department Functional Interfaces, Albert-Einstein-Strasse 9, 07745 Jena, Germany*

[2] *Institute of Physical Chemistry, Friedrich Schiller University Jena, Helmholtzweg 4, 07743 Jena, Germany*

[3] *Department of Engineering Physics, Institut Teknologi Sepuluh Nopember, Jl. Arief Rahman Hakim, 60111 Surabaya, Indonesia*

[4] *Institute of Inorganic Chemistry I, Ulm University, Albert-Einstein-Allee 11, 89081 Ulm, Germany*

[5] *Univ. Grenoble Alpes, CNRS, CEA, IRIG, Laboratoire de Chimie et Biologie des Métaux, 17 rue des Martyrs, F-38000 Grenoble, France*

[6] *Laboratory of Organic and Macromolecular Chemistry (IOMC), Friedrich Schiller University Jena, Humboldtstrasse 10, 07743 Jena, Germany*

[7] *Center for Energy and Environmental Chemistry Jena (CEEC Jena), Friedrich Schiller University Jena, Philosophenweg 7a, 07743 Jena, Germany*

[*] Corresponding authors e-mail: benjamin.dietzek@leibniz-ipht.de

---

Table of Content **Page**

1. **Synthesis and preparation of PPV, Ru's, and PPV-Ru's** **2**
   1.1. Materials and instrumentation 2
   1.2. Synthesis and characterization of the ruthenium complexes **Ru1** and **Ru2** 2
   1.3. Synthesis and characterization of the monomers 3
   1.4. Synthesis and characterization of the **PPV** polymer 6
   1.5. Synthesis and characterization of the **PPV-Ru1** and **PPV-Ru2** polymers 7
2. **UV/vis absorption and emission of PPV, Ru's, PPV-Ru's and simulated PPV-Ru's** **9**
3. **Electrochemistry and Spectroelectrochemistry Measurements** **10**
4. **Transient Absorption Study in Solution and on Film** **13**
5. **Photoelectrochemical Measurement** **23**
6. **Photostability Test of PPV-Ru System** **23**

1. Synthesis and Preparation of PPV, Ru's, and PPV-Ru's

1.1 Materials and Instrumentation

All chemicals and solvents were purchased from Sigma-Aldrich, Alfa Aesar as well as TCI and were used without further purification. NMR spectra were recorded on a 300 MHz NMR spectrometer (Fourier 300, Bruker Daltonics) or on a 400 MHz NMR spectrometer (Avance III HD 400, Bruker Daltonics) at 298 K and in deuterated solvents. Chemical shifts are reported in parts per million (ppm, δ scale) relative to the residual signal of the deuterated solvent. HR-MALDI-ToF-MS was performed at Ulm University using a Fourier Transform Ion Cyclotron Resonance (FT- ICR) mass spectrometer solariX (Bruker Daltonik) equipped with a 7.0 T superconducting magnet and interfaced to an Apollo II Dual ESI/MALDI source using trans-2-[3-(4-tert-butylphenyl)-2-methyl-2-propenylidene]malononitrile (DCTB) as a matrix. Elemental analyses were performed on a EuroVector EuroEA3000 elemental analyzer. The reaction progress was monitored by thin layer chromatography (TLC) using pre-coated aluminum sheets (silica gel 60 F254, Merck). Preparative column chromatography was performed on silica gel (pore size 60 Å, 70–230 mesh, 63–200 µm). Size exclusion chromatography (SEC) measurements of the copolymers were performed using a Shimadzu CBM-20A, DGU-14A as degasser, LC-20AD as the pump, a CTO-10AC vp oven with 40 °C oven temperature, a SPD-20A UVD-detector, a RID-10A RI-detector, a PSS SDV guard/1,000 Å/1,000,000 Å (5 µm particle size) column set, a flow rate of 1 mL/min, chloroform/*iso*-propanol/triethylamine (94/2/4) as the eluent and a polystyrene calibration. Preparative size exclusion chromatography was performed using Bio Beads® (S-X1, eluent: chloroform). IR measurements were performed on a Shimadzu IRAffinity-1.

1.2 Synthesis and characterization of the ruthenium complexes

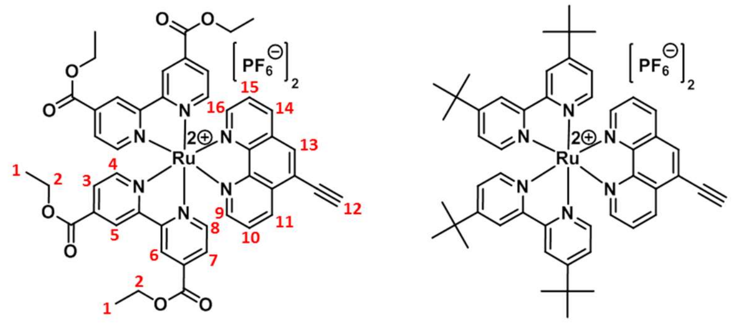

Scheme S1. Structures of ruthenium complexes **Ru1** and **Ru2**.

Ru2, $(Dceb)_2RuCl_2$ and 5-ethinyl-1,10-phenanthroline were prepared according to literature.[1,2]

[(4,4'-bis(ethylcarboxy)-2,2'-bipyridine)$_2$*(*5-ethinyl-1,10-phenanthroline*)ruthenium(II)](PF*$_6$*)*$_2$ *(***Ru1***)*

$(Dceb)_2RuCl_2$ (150 mg, 194 µmol) and 5-ethinyl-1,10-phenanthroline (41.7 mg, 204 µmol) were dissolved in a mixture of 40 mL ethanol and 10 mL H_2O and heated in the microwave for 1.5 h under reflux (130 W). The

solvent was removed under vacuum and an aqueous solution of 200 mg (1.23 mmol) NH_4PF_6 was added. The solid was filtered off, washed with water and small amounts of diethylether. After preparative column chromatography (silica gel, acetonitrile/H_2O/KNO_3, 90/10/0 → 90/9/1 v/v/v),precipitation in an aqueous NH_4PF_6-solution and filtering the ruthenium complex **Ru1** was obtained as a red solid (90.0 mg, 75.3 µmol, 39%).

^{1}H-NMR (400 MHz, CD_3CN, δ): 9.13-9.07 (m, 2H, H_6), 9.07-9.04 (m, 2H, H_5), 8.91 (dd, ^3J = 8.4 Hz, ^4J = 1.2 Hz, 1H, H_{11}), 8.64 (dd, ^3J = 8.3 Hz, ^4J = 1.2 Hz, 1H, H_{14}), 8.51 (s, 1H, H_{13}), 8.13 (dd, ^3J = 5.2 Hz, ^4J = 1.2 Hz, 1H, H_9), 8.09 (dd, ^3J = 5.3 Hz, ^4J = 1.2 Hz, 1H, H_{16}), 8.05 (d, ^3J = 5.8 Hz, 2H, H_8), 7.88-7.73 (m, 6H, H_7;H_4;H_{10};H_{15}), 7.71-7.64 (m, 2H, H_3), 4.51-4.35 (m, 8H, H_2), 4.18 (s, 1H, H_{12}), 1.46-1.33 (m, 12H, H_1) ppm.

^{13}C-NMR (101 MHz, CD_3CN, δ): 163.13, 163.07, 157.46, 157.07, 157.05, 153.24, 153.20, 153.18, 153.02, 152.98, 152.90, 146.68, 146.62, 138.96, 138.84, 137.12, 135.50, 132.50, 130.64, 130.09, 126.47, 126.43, 126.14, 126.10, 123.46, 123.43, 123.37, 120.26, 86.62, 77.53, 62.39, 62.34, 13.10, 13.06 ppm.

HR-MALDI-ToF-MS: m/z calculated for $[C_{46}H_{40}N_6O_8Ru]^+$: m/z = 906.19456, found: m/z = 906.19361$[M]^+$.

1.3 Synthesis and characterization of the monomers

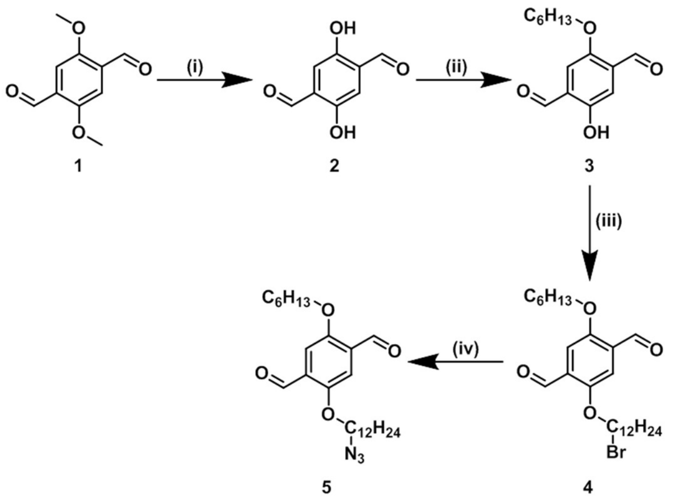

Scheme S2. Schematic representation of the synthetic procedure of monomer **5**: (i) Hydrobromic acid (48%)/glacial acetic acid/120 °C; (ii) 1-bromohexane/potassium carbonate/*N*,*N*-dimethyl-formamide/50 °C; (iii) 1,12-dibromododecane/potassium carbonate/*N*,*N*-dimethylformamide/55 °C; (iv) sodium azide/*N*,*N*-dimethylformamide/ 60 °C.

*2,5-Dihydroxyterephthalaldehyde (**2**)*

2,5-Dimethoxyterephthalaldehyde (**1**) (5.00 g, 25.75 mmol) was dissolved in 200 mL hydrobromic acid (48%) and 250 mL glacial acetic acid and purged with nitrogen for about 30 minutes. Afterwards, the resulting reaction mixture was heated to 120 °C overnight. After cooling to room temperature, the reaction mixture was poured into 800 mL of distilled water. After two hours the precipitate was filtered and washed ten times with 50 mL distilled water. The resulting solid was dried under vacuum and compound **2** was obtained as a dark yellow solid with a yield of 3.54 g (83%). The resulting compound **2** was used without further purification.

*2-(Hexyloxy)-5-hydroxyterephthalaldehyde (**3**)*

2,5-Dihydroxyterephthalaldehyde (**2**) (1.70 g, 10.23 mmol) and potassium carbonate (2.12 g, 15.35 mmol) were dissolved in 35 mL anhydrous *N,N*-dimethylformamide under nitrogen atmosphere. After adding 1-bromohexane (1.15 mL, 8.18 mmol) in one portion *via* a syringe, the reaction mixture was heated at 50 °C overnight. The reaction mixture was cooled to room temperature and, subsequently, 100 mL of distilled water and 5 mL of glacial acetic acid were added. The resulting suspension was extracted two times with 50 mL ethyl acetate. The combined organic phases were washed with 50 mL distilled water. After drying over sodium sulfate, the solvent was removed under vacuum. The resulting crude product was purified using preparative column chromatography (silica gel) with chloroform:methanol (99:1 vol%) as eluent. Compound **3** was obtained as a yellow solid with a yield of 0.78 g (38%).

^{1}H NMR (300 MHz, $CDCl_3$, δ): 10.52 (s, 1H, phenyl-C*H*O), 10.41 (s, 1H, phenyl-C*H*O), 9.97 (s, 1H, phenyl-O*H*), 7.42 (s, 1H, phenyl), 7.16 (s, 1H, phenyl), 4.09 (t, J = 6.3 Hz, 2H, O-CH_2), 1.87 (m, 2H, O-CH_2-CH_2), 1.60-1.25 (m, 6H, -CH_2), 0.92 (t, J = 7.1 Hz, 3H, -CH_3) ppm.

^{13}C NMR (75 MHz, $CDCl_3$, δ): 196.0, 189.1, 154.8, 154.0, 130.9, 123.7, 117.0, 116.2, 69.4, 31.5, 29.0, 25.7, 22.5, 14.0 ppm.

Elemental analysis: calcd. for $C_{14}H_{18}O_4$: C 67.18, H 7.25; found: C 67.45, H 7.28.

2-((12-Bromododecyl)oxy)-5-(hexyloxy)terephthalaldehyde (4)

2-(Hexyloxy)-5-hydroxyterephthalaldehyde (**3**) (0.70 g, 2.80 mmol), 1,12-dibromododecane (2.75 g, 8.39 mmol) and potassium carbonate (1.16 g, 8.39 mmol) were dissolved in 15 mL anhydrous *N,N*-dimethylformamide under nitrogen atmosphere. Afterwards, the reaction mixture was heated to 55 °C for four hours. After cooling to room temperature 100 mL of distilled water and 100 mL of ethyl acetate was added. The organic phase was washed with 50 mL distilled water two times and, afterwards, with 50 mL brine. The organic solvent was removed under vacuum. The crude product was purified *via* preparative column chromatography (silica gel) with chloroform as eluent. Compound **4** was obtained as a yellow solid with a yield of 1.13 g (82%).

^{1}H NMR (300 MHz, $CDCl_3$, δ): 10.52 (s, 2H, phenyl-C*H*O), 7.43 (s, 2H, phenyl), 4.09 (t, J = 6.4 Hz, 4H, O-CH_2), 3.41 (t, J = 6.8 Hz, 2H, Br-CH_2), 1.83 (m, 6H, O-CH_2-CH_2, Br-CH_2-CH_2), 1.60-1.15 (m, 22H, -CH_2), 0.91 (t, J = 7.0 Hz, 3H, -CH_3) ppm.

^{13}C NMR (75 MHz, $CDCl_3$, δ): 189.4, 155.2, 129.2, 111.6, 69.2, 34.0, 32.8, 31.4, 29.5, 29.4, 29.3, 29.0, 28.7, 28.1, 26.0, 25.7, 22.5, 14.0 ppm.

Elemental analysis: calcd. for $C_{26}H_{41}BrO_4$: C 62.77, H 8.31, Br 16.06; found: C 62.73, H 8.46, Br 16.37.

*2-((12-Azidododecyl)oxy)-5-(hexyloxy)terephthalaldehyde (**5**)*

2-((12-Bromododecyl)oxy)-5-(hexyloxy)terephthalaldehyde (**4**) (0.80 g, 1.61 mmol) and sodium azide (0.31 g, 4.82 mmol) were dissolved in anhydrous *N,N*-dimethylformamide under nitrogen atmosphere. Subsequently, the reaction mixture was heated to 60 °C overnight. After cooling to room temperature 100 mL of distilled water and 100 mL of ethyl acetate were added to the resulting reaction mixture. The aqueous phase was extracted two times with 50 mL ethyl acetate. The combined organic phases were washed two times with 50 mL of distilled water and 50 mL of brine. Afterwards, the solvent was removed under reduced pressure and the resulting crude

product was purified *via* column chromatography (silica gel) with chloroform as eluent. Compound **5** was obtained as a yellow solid with a yield of 0.72 g (97%).

^{1}H NMR (300 MHz, $CDCl_3$, δ): 10.52 (s, 2H, phenyl-C*H*O), 7.43 (s, 2H, phenyl), 4.09 (t, J = 6.4 Hz, 4H, O-CH_2), 3.26 (t, J = 6.9 Hz, 2H, N_3-CH_2), 1.83 (m, 4H, O-CH_2-CH_2), 1.70-1.20 (m, 24H, -CH_2), 0.91 (t, J = 7.0 Hz, 3H, -CH_3) ppm.

^{13}C NMR (75 MHz, $CDCl_3$, δ): 189.4, 155.2, 129.2, 111.6, 69.2, 51.5, 31.4, 29.5, 29.4, 29.3, 29.1, 29.0, 28.8, 26.7, 26.0, 25.6, 22.5, 14.0 ppm.

Elemental analysis: calcd. for $C_{26}H_{41}N_3O_4$: C 67.94, H 8.99, N 9.14; found: C 68.02, H 9.22, N 9.62.

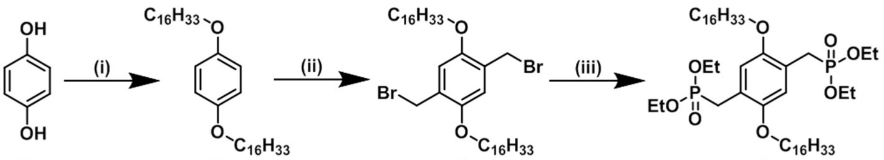

6 7 8 9

Scheme S3. Schematic representation of the synthetic procedure of monomer **9**: (i) 1-Bromohexadecane/potassium carbonate/ *N*,*N*-dimethylformamide/55 °C; (ii) Paraformaldehyde/sodium bromide/sulfuric acid/acetic acid/70 °C; (iii) Triethyl phosphite/150 °C.

1,4-Bis(hexadecyloxy)benzene (7)

Hydroquinone (**6**) (3.00 g, 27.24 mmol) and potassium carbonate (11.30 g, 81.74 mmol) were dissolved in 80 mL anhydrous *N*,*N*-dimethylformamide under nitrogen atmosphere. Subsequently, 1-bromohexadecane (26.13 mL, 81.74 mmol) was added *via* a syringe and the reaction mixture was heated at 55 °C overnight. Afterwards, the reaction mixture was poured into 600 mL distilled water and stirred for 30 minutes. The resulting precipitate was filtered and dried under reduced pressure. The crude product was purified *via* recrystallization in tetrahydrofuran. Compound **7** was obtained as a white solid with a yield of 10.42 g (68%).

^{1}H NMR (300 MHz, $CDCl_3$, δ): 6.83 (s, 4H, phenyl), 3.90 (t, J = 6.5 Hz, 4H, O-CH_2), 1.76 (m, 4H, O-CH_2-CH_2), 1.55-1.15 (m, 52H, -CH_2), 0.89 (t, J = 6.9 Hz, 6H, -CH_3) ppm.

^{13}C NMR (75 MHz, $CDCl_3$, δ): 153.2, 115.4, 68.6, 31.9, 29.7, 29.6, 29.4, 26.0, 22.7, 14.1 ppm.

Elemental analysis: calcd. for $C_{38}H_{70}O_2$: C 81.65, H 12.62; found: C 81.01, H 12.59.

*1,4-Bis(bromomethyl)-2,5-bis(hexadecyloxy)benzene (**8**)*

1,4-*Bis*(hexadecyloxy)benzene (**7**) (8.00 g, 14.31 mmol), paraformaldehyde (6.02 g, 200,36 mmol) and sodium bromide (7.80 g, 75.84 mmol) were dissolved in 100 mL glacial acetic acid. After the reaction mixture was heated to 60 °C a mixture of 15.3 g concentrated sulfuric acid and 15.3 g glacial acetic acid was added dropwise *via* a syringe. Afterwards, the suspension was heated at 70 °C for four hours. The reaction mixture was slowly cooled to 0 °C and the resulting precipitate was filtered and washed ten times with 50 mL distilled water. The crude product was dried under vacuum and further purified *via* column chromatography (silica gel) with chloroform as eluent. Compound **8** was obtained as a white solid with a yield of 3.91 g (37%).

^{1}H NMR (300 MHz, $C_6D_4Cl_2$, 50 °C, δ): 6.86 (s, 2H, phenyl), 4.59 (s, 4H, Br-CH_2) 3.90 (t, J = 6.2 Hz, 4H, O-CH_2), 1.82 (m, 4H, O-CH_2-CH_2), 1.60-1.15 (m, 52H, -CH_2), 0.95 (t, J = 6.9 Hz, 6H, -CH_3) ppm.

^{13}C NMR (75 MHz, $C_6D_4Cl_2$, 50 °C, *δ*): 151.7, 115.5, 69.9, 33.0, 30.8, 30.5, 29.6, 27.2, 23.8, 15.1 ppm.

Elemental analysis: calcd. for $C_{40}H_{72}Br_2O_2$: C 64.50, H 9.74, Br 21.46; found: C 65.01, H 9.88, Br 21.62.

*Tetraethyl ((2,5-bis(hexadecyloxy)-1,4-phenylene)bis(methylene))bis(phosphonate) (**9**)*

1,4-*Bis*(bromomethyl)-2,5-*bis*(hexadecyloxy)benzene (**8**) (3.50 g, 4.70 mmol) and triethyl phosphite (3.27 mL, 18.80 mmol) was heated at 150 °C for four hours. Afterwards, the reaction mixture was heated 30 minutes to 180 °C under vacuum in order to remove the excess of the triethyl phosphite. After cooling to room temperature the crude product was purified *via* recrystallization in diethyl ether. Compound **9** was obtained as a white solid with a yield of 3.10 g (77%).

^{1}H NMR (300 MHz, $CDCl_3$, *δ*): 6.91 (s, 2H, phenyl), 4.02 (m, 8H, O-CH_2), 3.91 (t, J = 6.5 Hz, 4H, O-CH_2), 3.25 (s, 2H, P-CH_2), 3.19 (s, 2H, P-CH_2), 1.76 (m, 4H, O-CH_2-CH_2), 1.60-1.10 (m, 64H, -CH_2, -P-CH_2-CH_3), 0.88 (t, J = 6.9 Hz, 6H, -CH_3) ppm.

^{13}C NMR (75 MHz, $CDCl_3$, *δ*): 150.4, 119.4, 114.9, 69.0, 61.8, 31.9, 29.7, 29.6, 29.4, 29.3, 26.1, 22.7, 16.3, 14.1 ppm.

Elemental analysis: calcd. for $C_{48}H_{92}O_8P_2$: C 67.10, H 10.79; found: C 67.68, H 10.94.

1.4 Synthesis and characterization of the PPV polymer

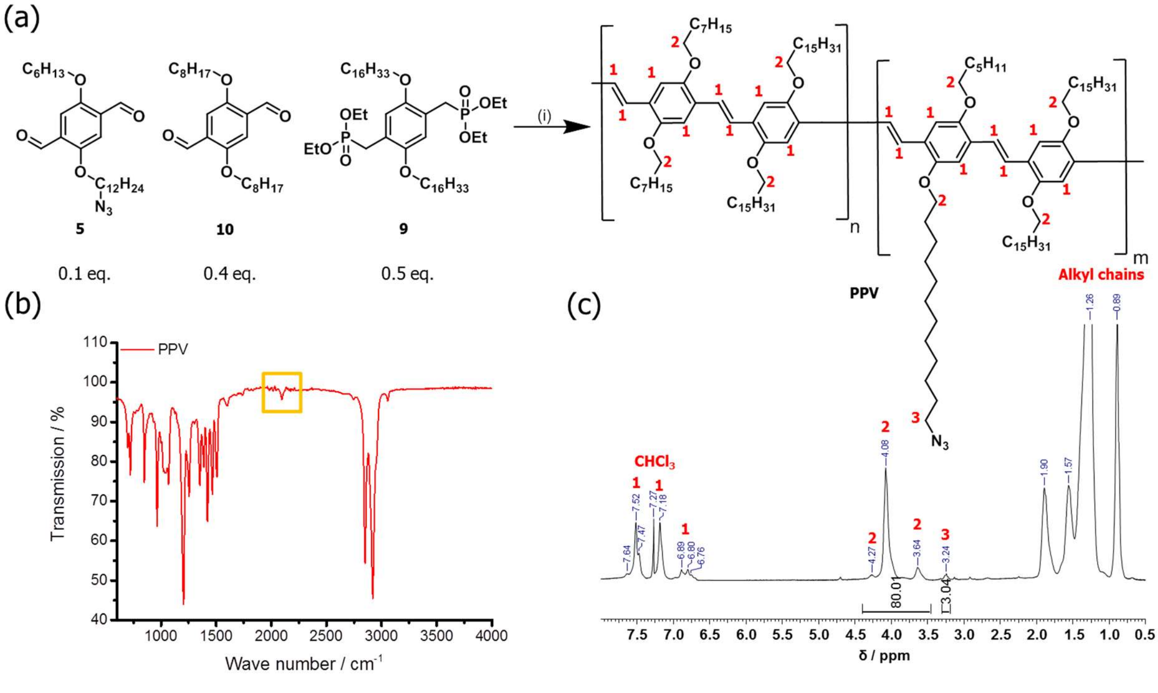

Figure S1. (a) Schematic representation of the synthetic procedure of the **PPV** polymer: (i) Potassium *t*-butoxide/*N*,*N*-dimethylformamide/100 °C; (b) IR spectrum of the **PPV** polymer; (c) ^{1}H NMR spectrum of the **PPV** polymer.

Poly(**9**-*co*-**10**)-*stat*-(**9**-*co*-**5**) (**PPV**)

Tetraethyl ((2,5-*bis*(hexadecyloxy)-1,4-phenylene)*bis*(methylene))*bis*(phosphonate) (**9**) (220 mg, 0.256 mmol), 2-((12-azidododecyl)oxy)-5-(hexyloxy)terephthalaldehyde (**5**) (24 mg, 0.05 mmol), 2,5-

bis(octyloxy)terephthalaldehyde (80 mg, 0.205 mmol) and potassium *t*-butoxide (144 mg, 1.280 mmol) were dissolved in 5 mL anhydrous *N*,*N*-dimethylformamide under nitrogen atmosphere. The resulting reaction mixture was heated at 100 °C for 24 hours. After cooling to room temperature 60 mL of methanol was added. The precipitate was filtered and washed five times with 10 mL methanol, five times with 10 mL distilled water and five times with 10 mL acetone. After drying under vacuum the **PPV** polymer was obtained as a red solid with a yield of 202 mg (83%).

The degree of the azide functionalization of the **PPV** polymer was determined to be approximately 7.6% by ^{1}H NMR (Figure S1(c)).

Moreover, the IR spectrum (Figure S1(b)) of the **PPV** polymer show the characteristic peak at around 2100 cm^{-1} for the asymmetric stretching mode of the azide.

Parts of the polymer sample were analyzed by size exclusion chromatography (SEC: chloroform/*iso*-propanol/trimethylamine (94/2/4) as eluent, polystyrene as calibration) to determine an average molar mass M_n of 10,700 g/mol and a dispersity (Đ) of 2.33.

^{1}H NMR (300 MHz, $CDCl_3$, *δ*, Figure S1(a)): 7.80-6.60 (m, phenyl, phenyl-C*H*=), 4.50-3.40 (m, O-CH_2), 3.24 (m, N_3-CH_2), 2.10-0.70 (m, -CH_2, -CH_3) ppm.

Elemental analysis: Anal. calcd. for the repeating units and monomer ratio based on ^{1}H NMR: (Figure S1(c)): C 81.15, H 11.48, N 0.62; found: C 79.82, H 11.51, N 0.66.

1.5 Synthesis and characterization of the PPV-Ru1 and PPV-Ru2 polymers

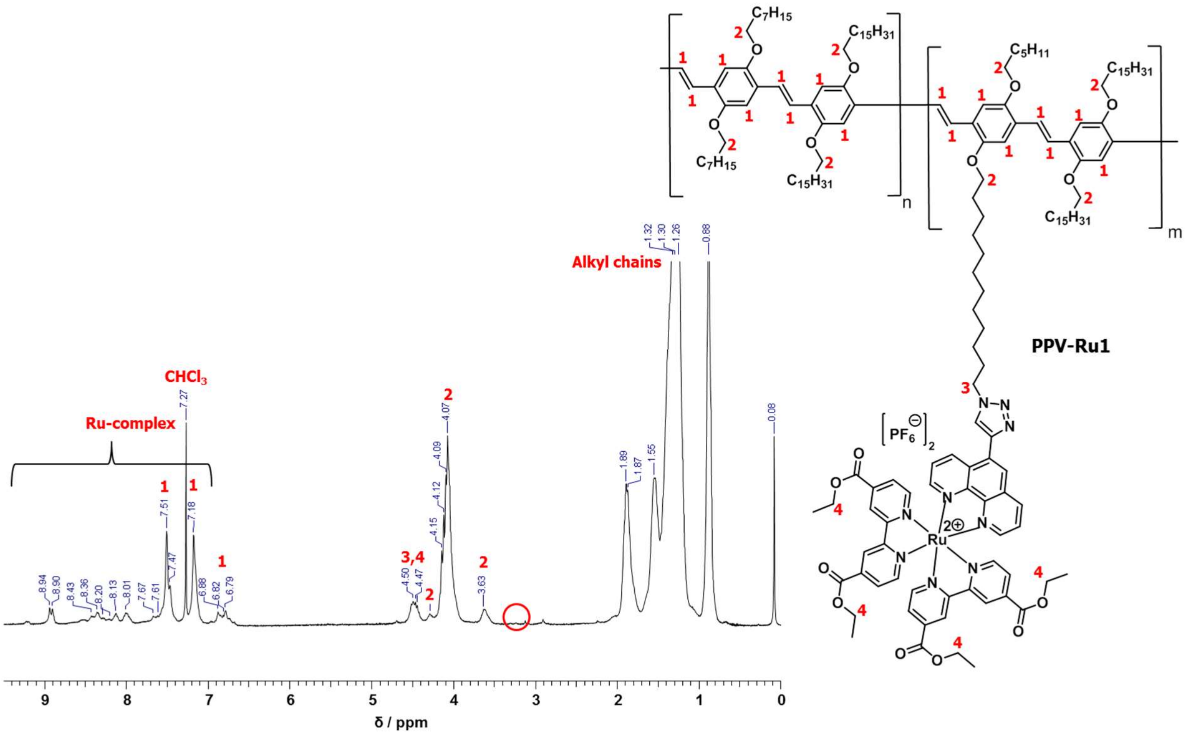

Figure S2. ^{1}H NMR spectrum of the **PPV-Ru1** polymer.

Poly(**9**-*co*-**10**)-*stat*-(**9**-*co*-(**5-Ru1**)) (**PPV-Ru1**)

Poly(**9**-*co*-**10**)-*stat*-(**9**-*co*-**5**) (**PPV**) (34.0 mg, 0.0054 mmol), Ru-complex **Ru1** (8.0 mg, 0.0069 mmol) and iodo(triethyl phosphite)copper(I) (4.8 mg, 0.0134 mmol) were dissolved in 0.4 mL anhydrous chloroform under nitrogen atmosphere. Subseqently, 0.93 µl anhydrous triethylamine was added *via* a syringe and the resulting reaction mixture was heated at 60 °C under exclusion of light overnight. The mixture was cooled to room temperature and precipitated in 20 mL of a methanol/saturated NH_4PF_6-solution (3:1 vol%). The precipitate was filtered and washed two times with 10 mL distilled water and two times with 10 mL methanol. Afterwards, the crude product was dried under vacuum and was further purified *via* preparative size exclusion chromatography (Biobeads S-X1) with chloroform as eluent. **PPV-Ru1** was obtained as a dark red solid with a yield of 36 mg (90%). The coupling degree of the **Ru1** functionalization with the azide functions of the **PPV** polymer was determined to be approximately 100% by ^{1}H NMR due to the disappearance of the signal at 3.24 ppm, which corresponds to the CH_2 group next to the azide function (Figure S2).

Parts of the polymer sample were analyzed by size exclusion chromatography (SEC: chloroform/*iso*-propanol/triethylamine (94/2/4) as eluent, polystyrene as calibration) to determine an average molar mass M_n of 10,600 g/mol and a dispersity (Đ) of 1.80.

^{1}H NMR (300 MHz, $CDCl_3$, δ, Figure S2): 9.40-6.60 (m, phenyl, phenyl-C*H*=, Ru-complex ligands of **Ru1**, triazole), 4.50 (m, O-CH_2 (Ru-complex), triazole-CH_2) 4.35-3.50 (m, O-CH_2 (phenyl)), 2.10-0.70 (m, -CH_2, -CH_3) ppm.

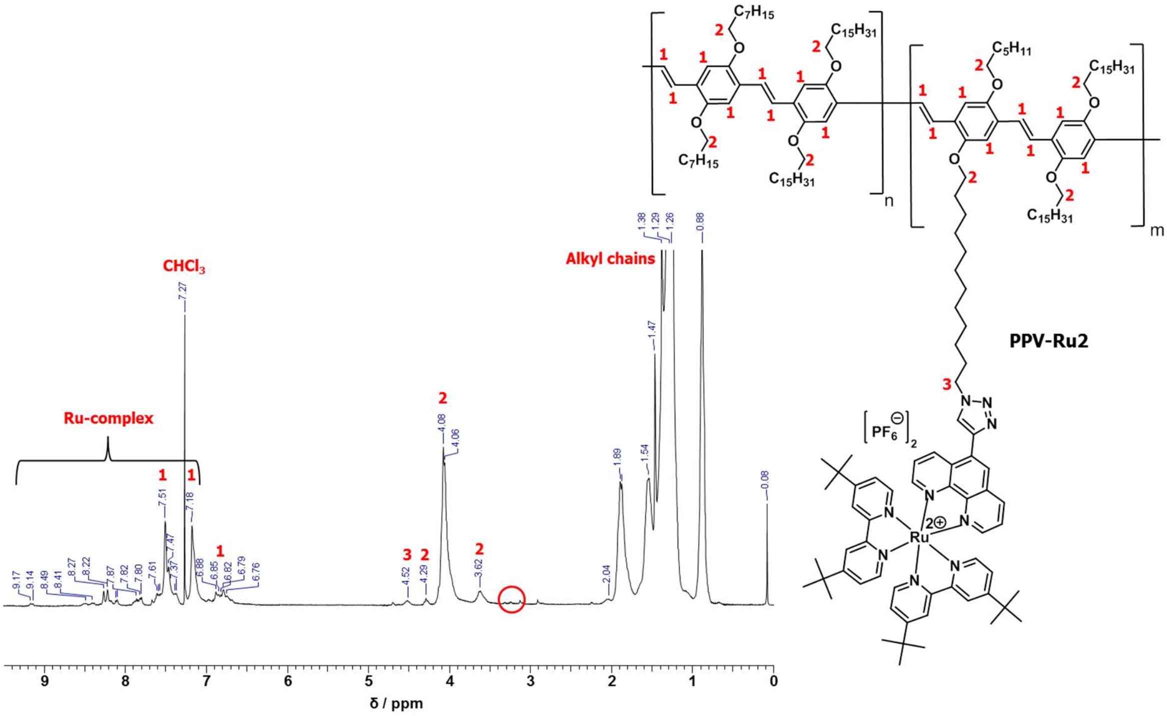

Figure S3. ^{1}H NMR spectrum of the **PPV-Ru2** polymer.

Poly(**9**-*co*-**10**)-*stat*-(**9**-*co*-(**5**-**Ru2**)) (**PPV-Ru2**)

Poly(**9**-*co*-**10**)-*stat*-(**9**-*co*-**5**) (**PPV**) (87.0 mg, 0.0138 mmol), Ru-complex **Ru2** (19.5 mg, 0.0172 mmol) and iodo(triethyl phosphite)copper(I) (12.3 mg, 0.0344 mmol) were dissolved in 0.8 mL anhydrous chloroform under

nitrogen atmosphere. Subseqently, 2.40 µl anhydrous triethylamine was added *via* a syringe and the resulting reaction mixture was heated at 60 °C under exclusion of light overnight. The mixture was cooled to room temperature and precipitated in 40 mL of a methanol/saturated NH_4PF_6-solution (3:1 vol%). The precipitate was filtered and washed two times with 20 mL distilled water and two times with 20 mL methanol. Afterwards, the crude product was dried under vacuum and was further purified *via* präparative size exclusion chromatography (Biobeads S-X1) with chloroform as eluent. **PPV-Ru2** was obtained as a dark red solid with a yield of 97 mg (92%). The coupling degree of the **Ru2** functionalization with the azide functions of the **PPV** polymer was determined to be approximately 100% by ^{1}H NMR due to the disappearance of the signal at 3.24 ppm, which corresponds to the CH_2 group next to the azide function (Figure S3).

Parts of the polymer sample were analyzed by size exclusion chromatography (SEC: chloroform/*iso*-propanol/triethylamine (94/2/4) as eluent, polystyrene as calibration) to determine an average molar mass M_n of 12,200 g/mol and a dispersity (Đ) of 2.07.

^{1}H NMR (300 MHz, $CDCl_3$, δ, Figure S3): 9.40-6.60 (m, phenyl, phenyl-CH=, Ru-complex ligands of **Ru1**, triazole), 4.52 (m, triazole-CH_2) 4.40-3.45 (m, O-CH_2 (phenyl)), 2.20-0.70 (m, -CH_2, -CH_3) ppm.

2. UV/vis Absorption and Emission of PPV-Ru, PPV, Ru, and Simulated PPV-Ru

The UV/vis absorption and emission spectra of **PPV-Ru** and simulated **PPV-Ru** (linear superposition of absorption and emission from the corresponding **PPV** and **Ru**) are compared (Figure S4a and Figure S4b). It is discussed in the main text that the emission contribution from the Ru photosensitizer is not observed in the emission spectrum of **PPV-Ru**: The emission spectrum of **PPV-Ru** resembles the emission spectrum of **PPV**. This might indicate that the emission of photosensitizer is quenched when attached to the **PPV** backbone. As emission quenching indicates hole injection, which is important in this work, further evidence is required. Emission quenching is supported from the simulated emission spectra, in which the emission spectrum of **PPV** is linearly superposed with the emission spectrum of **Ru**. The simulated results show the off-set in the spectral region 630 to 650 nm between the emission spectrum of **PPV** and simulated **PPV-Ru**, indicating that there should not be any quenching if there is no charge injection across **PPV –Ru**. Since there is no off-set in the spectral region of 630 to 650 nm between the emission spectra of **PPV** and **PPV-Ru**, Ru emission quenching occurs and hole injection is highly probable. In addition, the extinction absorption coefficients of **PPV** and **Ru1** as well as **PPV-Ru1** are presented in Figure S4c. It can be seen that the absorption of **Ru1** in the visible region is only a quarter of the absorption of **PPV**. Considering the low loading of Ru (7.6%) in **PPV-Ru**, the fraction of photons absorbed by **Ru** will only account for 1.5% of total photon absorbed in **PPV-Ru** if the system is irradiated by a visible light source, for example photoexcitation at 480 nm in the transient absorption study (*vide infra*).

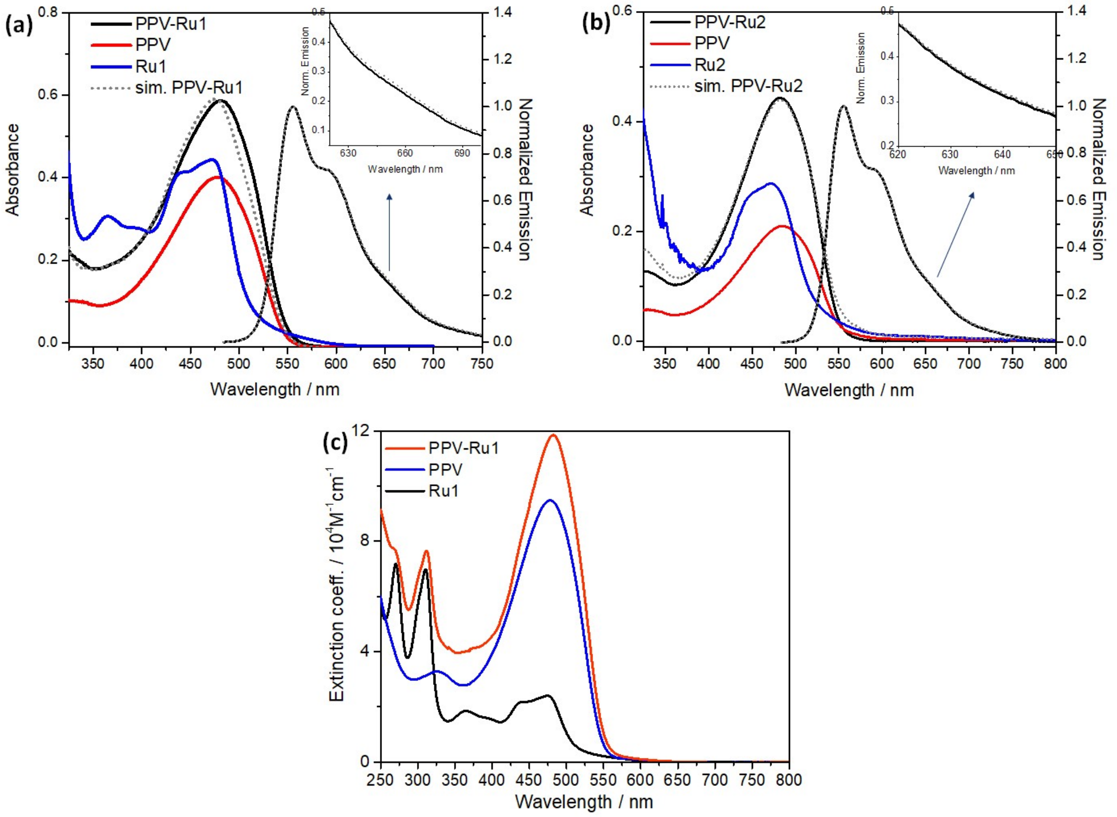

Figure S4. (a) UV/vis absorption spectra of **PPV-Ru1**, **PPV**, **Ru1**, measured in $CHCl_3$ and simulated **PPV-Ru1**. Emission spectra of **PPV-Ru1** and simulated **PPV-Ru1** are compared. (b) UV/vis absorption spectra of **PPV-Ru2**, **PPV**, **Ru2**, measured in $CHCl_3$ and simulated **PPV-Ru2**. Emission spectra of **PPV-Ru2** and simulated **PPV-Ru2** are compared. (c) The extinction coefficient of **PPV-Ru1**, **PPV**, and **Ru1**, measured in $CHCl_3$.

3. Electrochemistry and Spectroelectrochemistry Measurements

Electrochemical studies using cyclic voltammetry were carried out to obtain reduction and oxidation potentials of individual components of the **PPV-Ru** systems, which are used to estimate the driving force of possible charge injection processes.

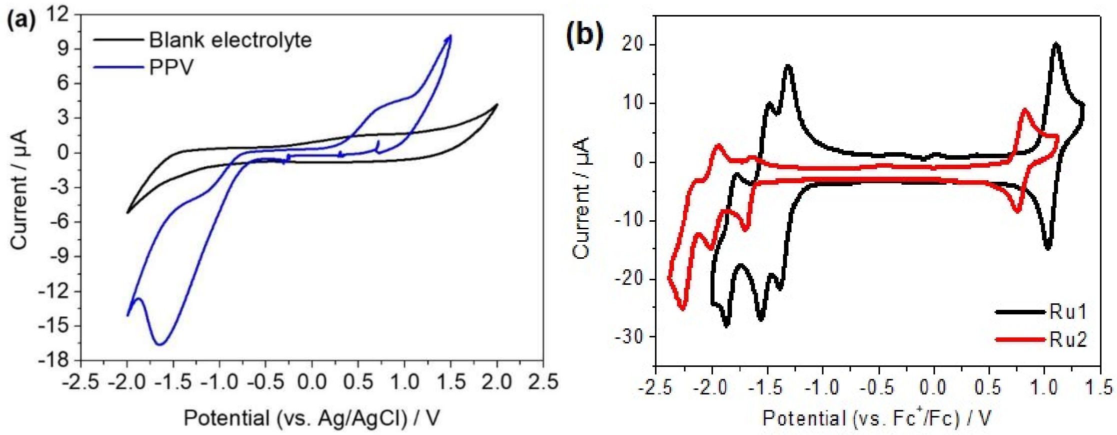

Figure S5. Electrochemical reduction and oxidation of (a) **PPV** measured in $CHCl_3$ and (b) **Ru1** and **Ru2** measured in acetonitrile. The supporting electrolyte was 0.1M $TBABF_4$ and the scan rate was 0.1 V/s. Inset of figure (b) indicates the current-voltage curve of the supporting electrolyte in acetonitrile.

Spectroelectrochemistry measurements were carried out to find the "optical tag" of transient species observed in transient absorption measurements.

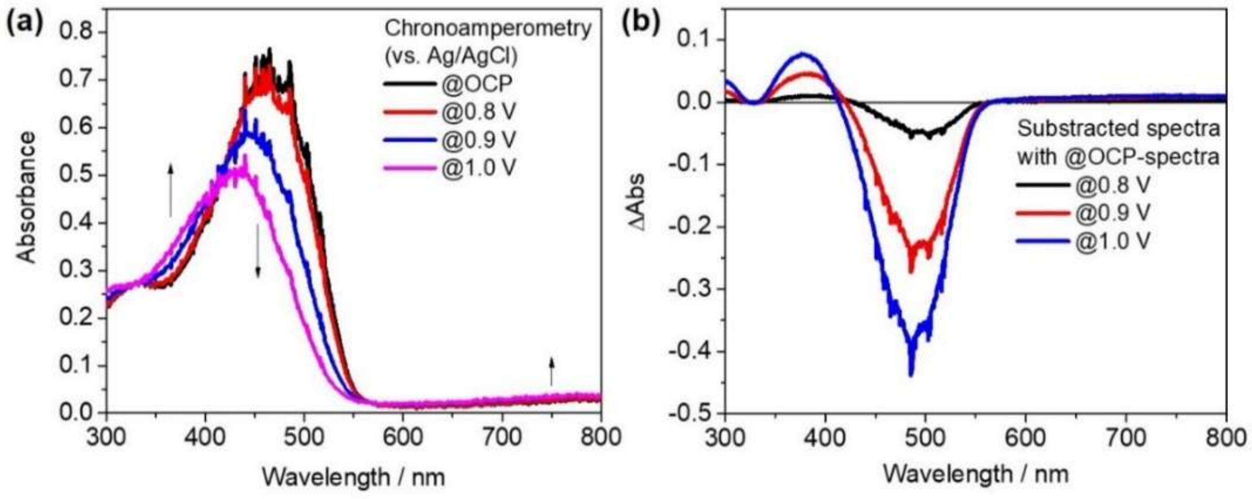

Figure S6. (a) Spectroelectrochemical UV/vis absorption spectra and (b) UV/vis absorption difference spectra of **PPV** collected in $CHCl_3$ by sequential oxidations in chronoamperometric measurements.

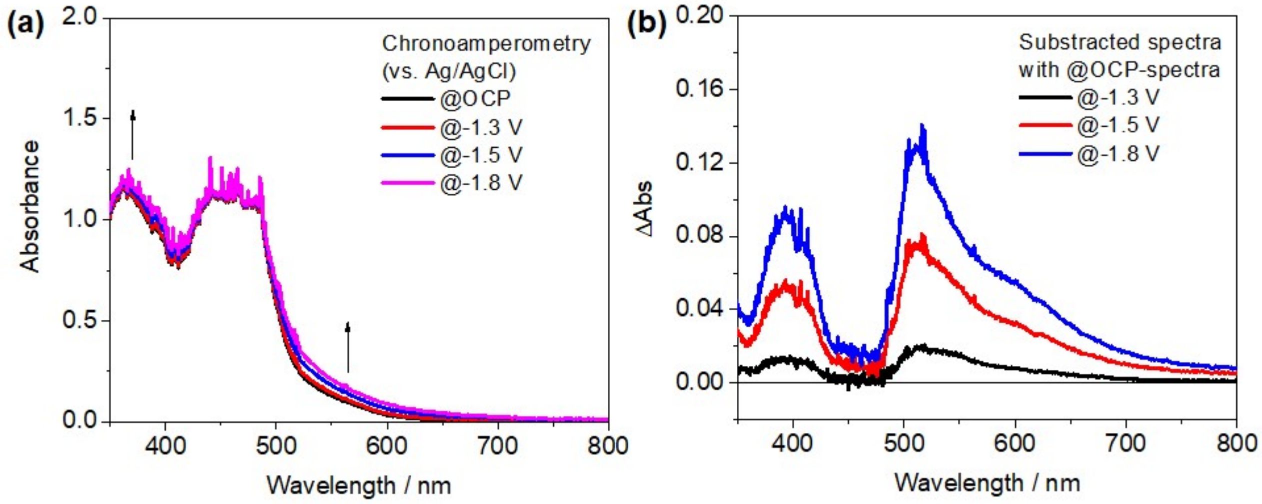

Figure S7. (a) Spectroelectrochemical UV/vis absorption spectra and (b) UV/vis absorption difference spectra of **Ru1** collected in $CHCl_3$ by sequential reductions in chronoamperometric measurements.

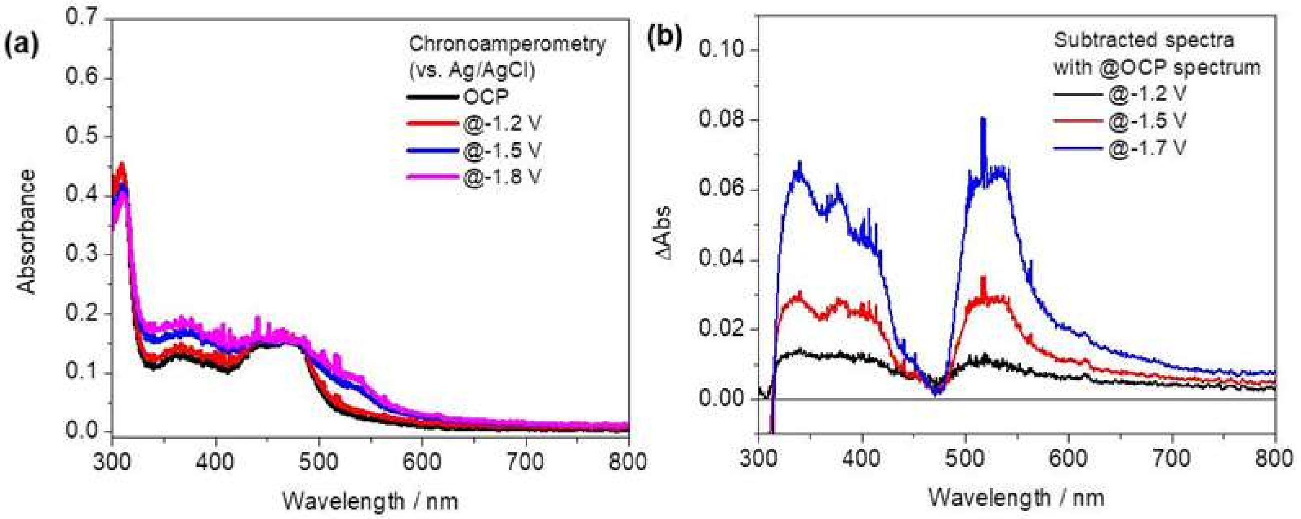

Figure S8. (a) Spectroelectrochemical UV/vis absorption spectra and (b) UV/vis absorption difference spectra of **Ru2** collected in $CHCl_3$ by sequential reductions in chronoamperometric measurements.

As the **PPV-Ru** system will be used as a photocathode, UV/vis spectroelectrochemistry (SEC) measurements of **PPV-Ru1** films were carried out to (again) find the "optical tag" of transient species, *i.e.* reduced diimine ligands (either bpy or phen ligand). Thin (~ 300 nm) and thick (~1.6 µm, Fig. S9) **PPV-Ru1** films were prepared for the SEC measurements. The reductive SEC spectra at the reduction potential of **Ru1** and the absorption difference spectra are depicted in Fig. S10. Compared to the spectra of reduced **Ru1** in solution, it is clear that the absorption difference peak at 390 and 530 nm is shifted to 425 and 575 nm, respectively.

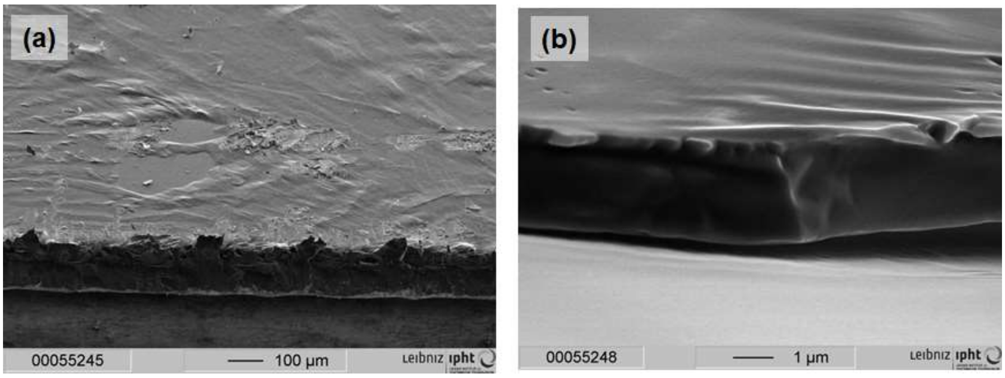

Figure S9. (a) SEM images showing the morphology of a **PPV-Ru1** film and (b) cross-section image showing the thickness of **PPV-Ru1** films (1.96 ± 0.61 µm).

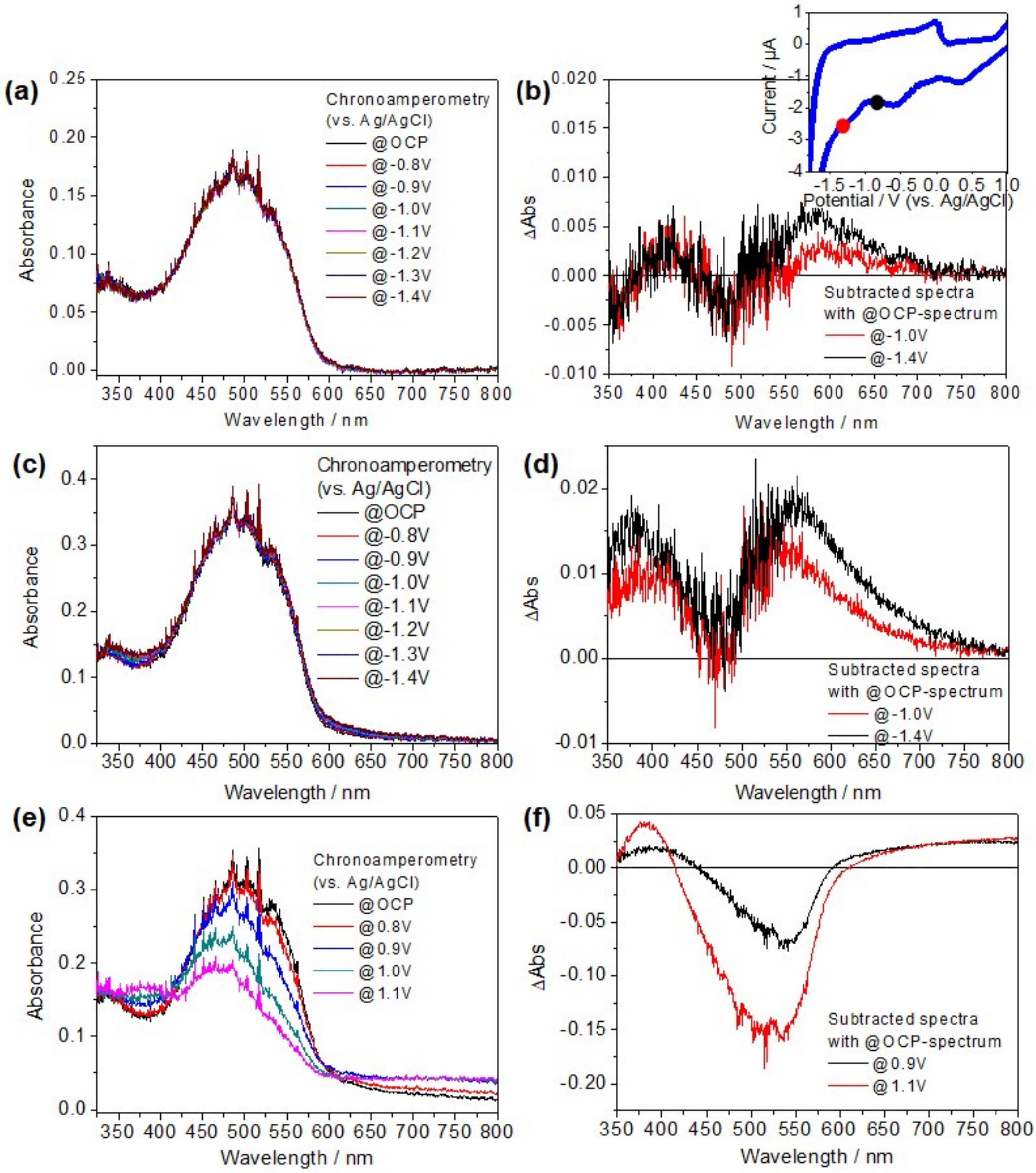

Figure S10. Spectroelectrochemical UV/vis absorption spectra and UV/vis absorption difference spectra of **PPV-Ru1** films by sequential reductions (a,b for thin film (d ~ 300 nm) and c,d for thick film (d~1.6 μm)) and oxidation (e,f) in chronoamperometric measurements. The inset of panel (b) indicates the cyclic voltammogram of **PPV-Ru1** thin films.

4. Transient Absorption Study in Solution and on Films

The fs-transient absorption (TA) study is used to unravel the light-induced charge transfer dynamics in **PPV-Ru** both in solution and on film. The transient absorption data of **PPV** as discussed in the main text show an initial ground-state bleach (GSB), stimulated emission (SE) at 530 (peak) and 565 nm (shoulder), and an excited-state absorption (ESA) band beyond 600 nm (Fig. S11a). Arbitrarily scaled absorption and emission spectra of PPV are also shown to discriminate GSB and SE contribution in the TA data. A global fit of the TA data using three decay components results in the decay-associated spectra shown in Fig. S11c. The spectral features associated with τ_1 show a negative ΔOD signal at 450 – 570 nm reflecting the decay of the excited state of PPV and is attributed to vibrational cooling.[3] The spectral signature of τ_2 is quite similar to τ_1 with a distinct peak at 520 nm. This process

(τ_2 = 72 ps) is assigned to interchain or intrachain energy transfer which typically falls on a characteristic timescale of tens of ps.[4] The following process associated with the time constant of hundreds of ps (τ_3) reflects a concerted decay of SE and absorption beyond 750 nm (spectral signature of radical cation/polaron $PPV^{\bullet+}$),[5,6] and hence, it is assigned to exciton recombination.

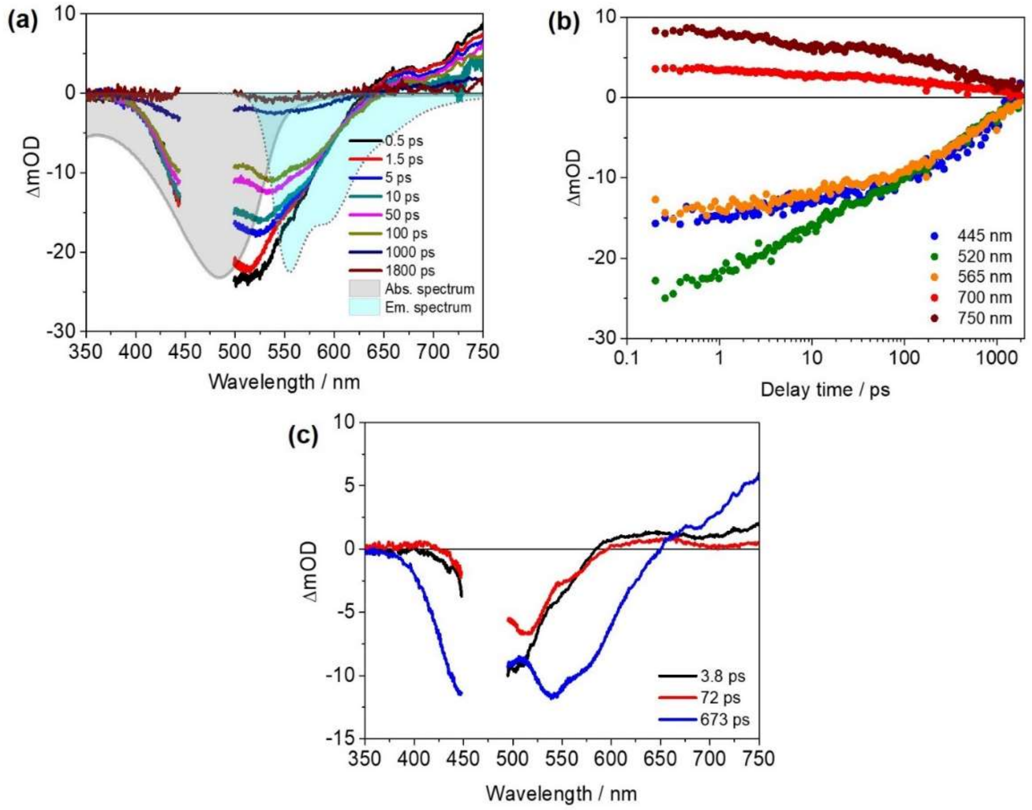

Figure S11. (a) fs-transient absorption spectra of **PPV** in $CHCl_3$ at different delay times, (b) kinetic traces at different probe wavelengths, and (c) decay-associated spectra resulting from the global fit with three exponential decay function. The grey area depicts the corresponding inverted ground state absorption spectrum of **PPV**, which is arbitrarily scaled to fit the transient absorption data.

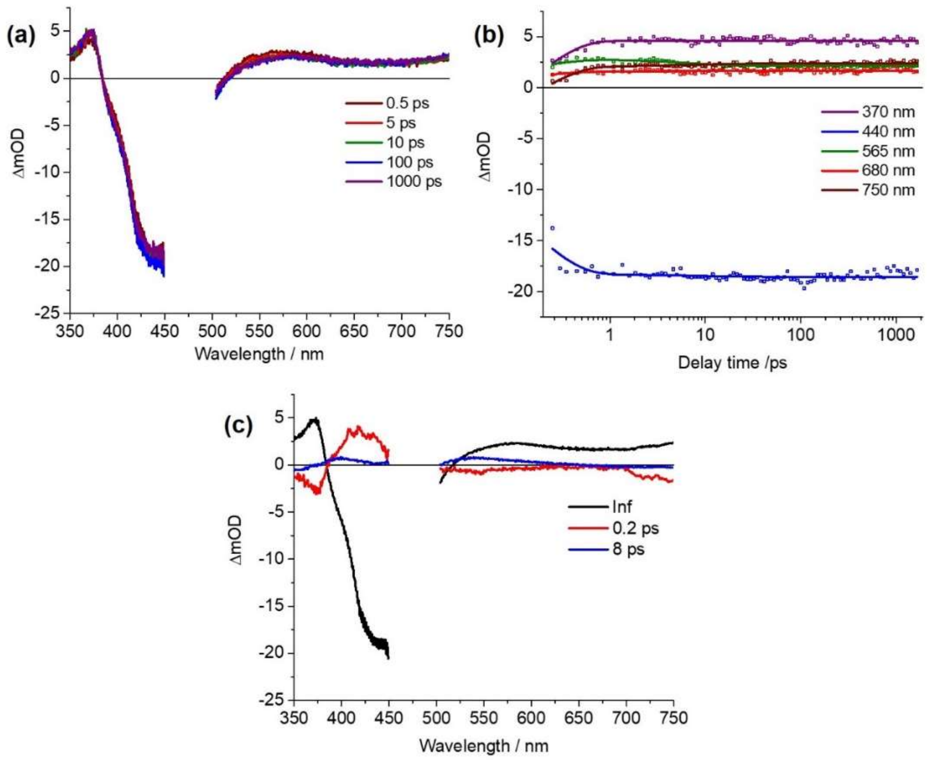

Figure S12. (a) fs-transient absorption spectra of **Ru1** in $CHCl_3$ at different delay times, (b) kinetic traces at different probe wavelengths, and (c) decay-associated spectra resulting from the global fit with two exponential decay function and an infinite component compensating the long-lived signal.

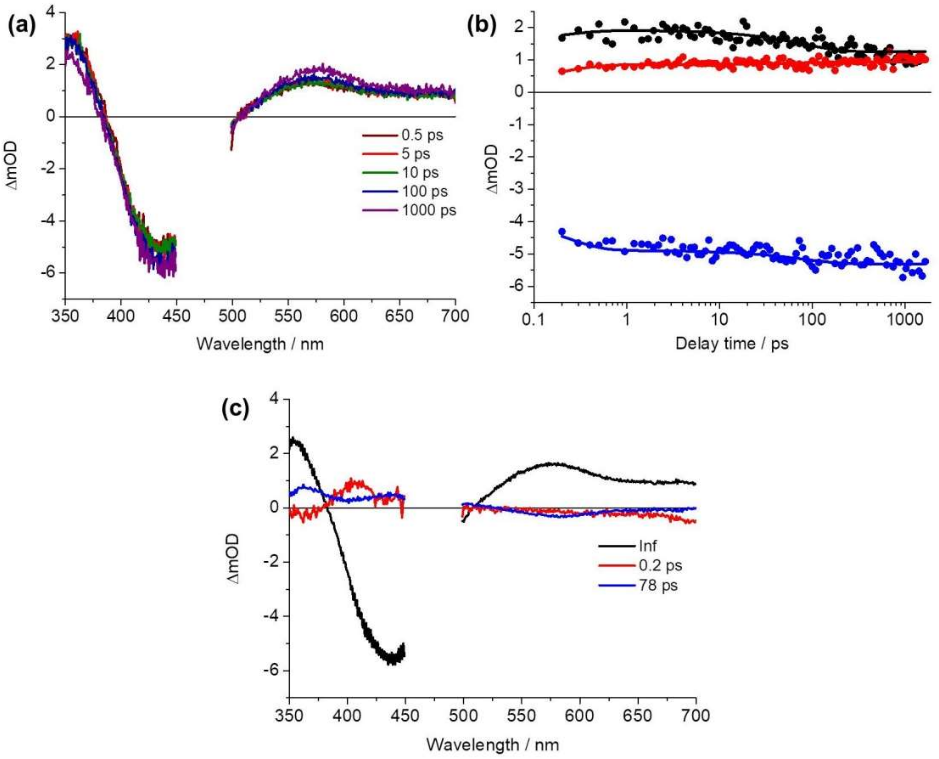

Figure S13. (a) fs-transient absorption spectra of **Ru2** in $CHCl_3$ at different delay times, (b) kinetic traces at different probe wavelengths, and (c) decay-associated spectra resulting from the global fit with two exponential decay function and an infinite component compensating the long-lived signal.

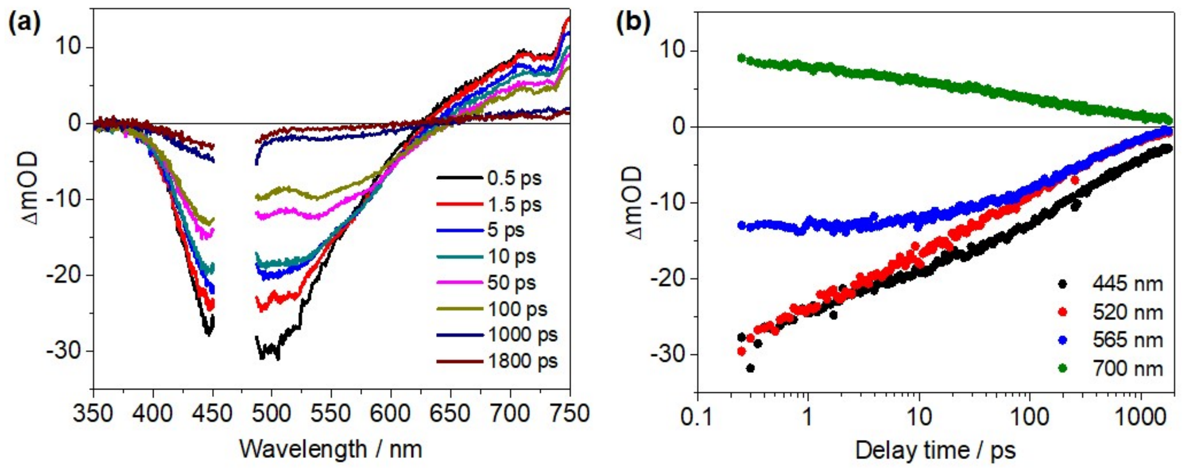

Figure S14. (a) fs-transient absorption spectra of **PPV-Ru1** in $CHCl_3$ at different delay times and (b) kinetic traces at different probe wavelengths.

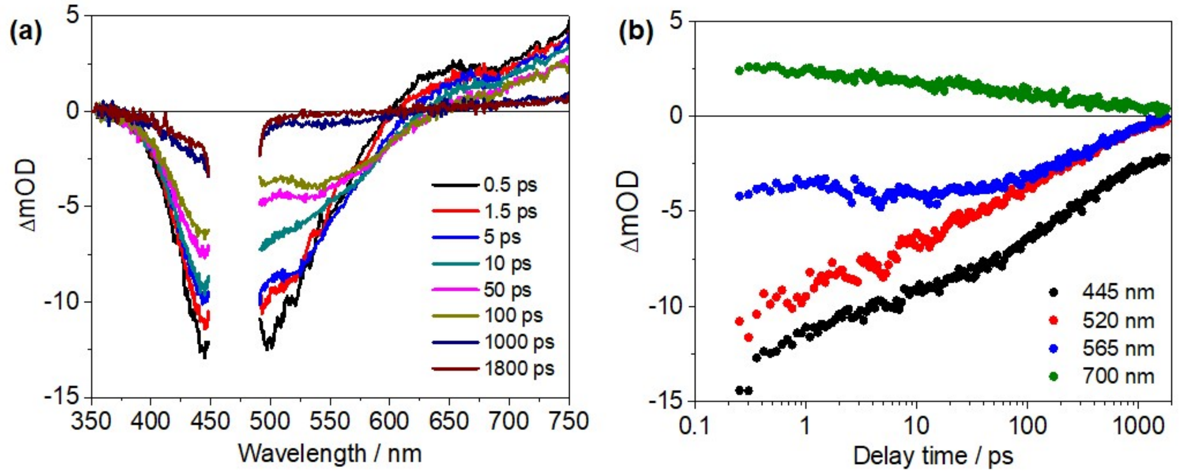

Figure S15. (a) fs-transient absorption spectra of **PPV-Ru2** in $CHCl_3$ at different delay times and (b) kinetic traces at different probe wavelengths.

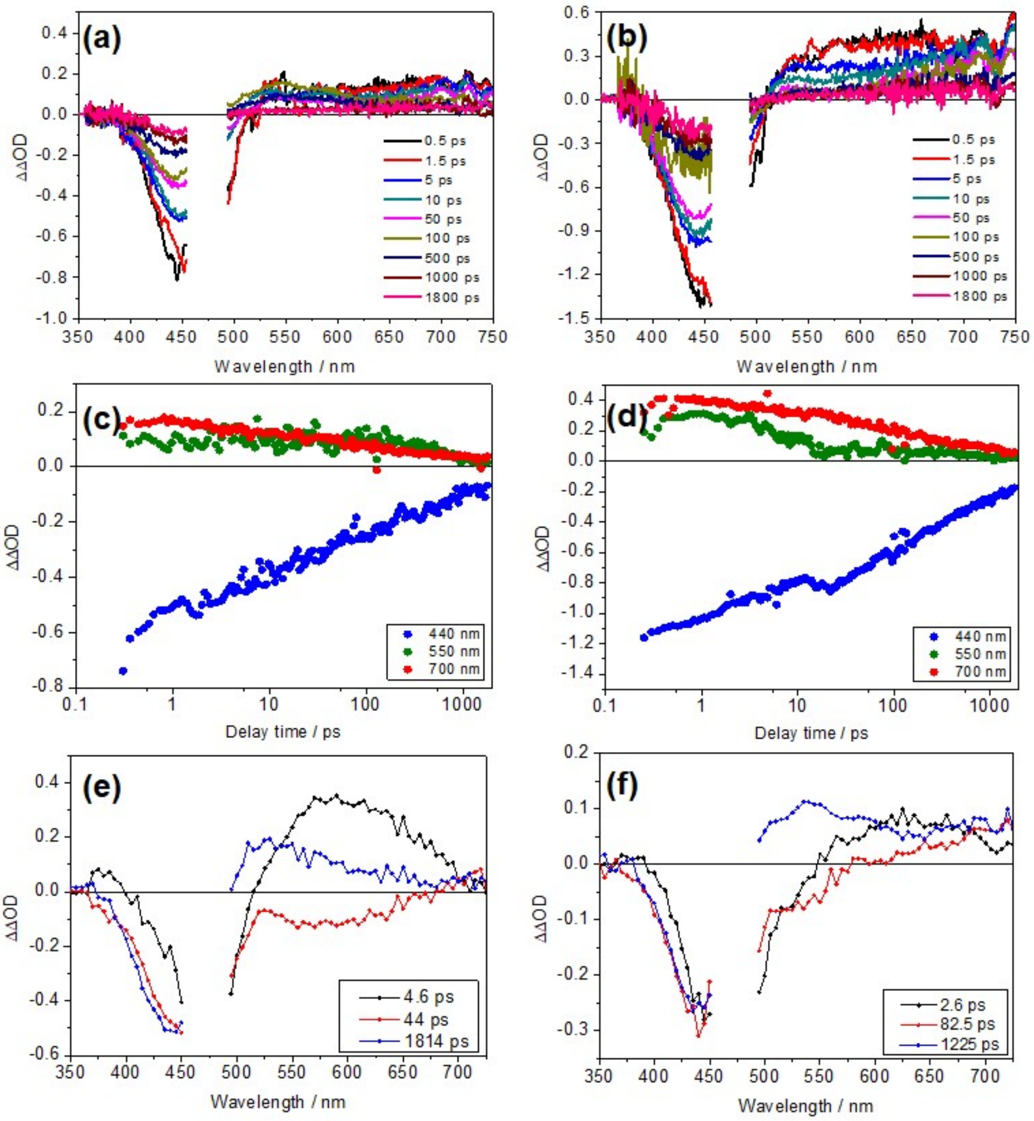

Figure S16. Differential fs-transient absorption spectra of (a) **PPV-Ru1** and (b) **PPV-Ru2** in $CHCl_3$ at different delay times. Normalized kinetic traces at different probe wavelengths and decay-associated spectra (DAS) resulting from the global fit with three time constants for (c,e) **PPV-Ru1** and (d,f) **PPV-Ru2**. The pump pulses are centred at 480 nm. The grey spectra in panel (e) and (f) depict the differential absorption spectra of electrochemically reduced **Ru1** and **Ru2**, respectively, which is arbitrarily scaled to fit the transient absorption data.

Table S1. Characteristic time constants (τ) from global fitting of differential fs-transient absorption data from three different measurements.

System	τ_1 / ps	τ_2 / ps	τ_3 / ps
**PPV-Ru1**	3.3 ± 1.2	49 ± 4	1587 ± 201
**PPV-Ru2**	4.0 ± 1.3	86 ± 9	994 ± 203

ns-transient absorption (TA) unravels the spectroscopic signatures of the long-lived species in **PPV** and **PPV-Ru** upon photoexcitation. The ns- TA data of **PPV** and **PPV-Ru1** are depicted in Figure S17a and Figure S18b, respectively. TA data of **PPV** and **PPV-Ru1** shows a spectral signature beyond 750 nm, which can be assigned to either ^{3}PPV or polaron.[5,6] The lifetime of this state is found to be > 40 µs, which is in good agreement with literature.[7,8] Furthermore, similar to the fs-TA data processing for **PPV-Ru** in solution, the data obtained for **PPV** alone is then

subtracted from the ns-TA signal of **PPV-Ru1** (Figure S17c). Prior to subtraction the data is normalized to its early time (Δt = 200 ns) signal amplitude at 520 nm for **PPV-Ru1** at which there is no contribution of the transient absorption signal from **Ru** alone. Apparently, the resulting ΔΔOD spectra in Figure S17c show no signal even at the early time scale of 200 ns. This result indicates that the charge separation process in **PPV-Ru**1 occurs in a few ns as revealed in fs-TA study. Therefore, the long-lived signal in **PPV-Ru1** might root from the ^{3}PPV signal of the fragment of **PPV-Ru1**, in which the polymer backbone isn't loaded with the photosensitizers.

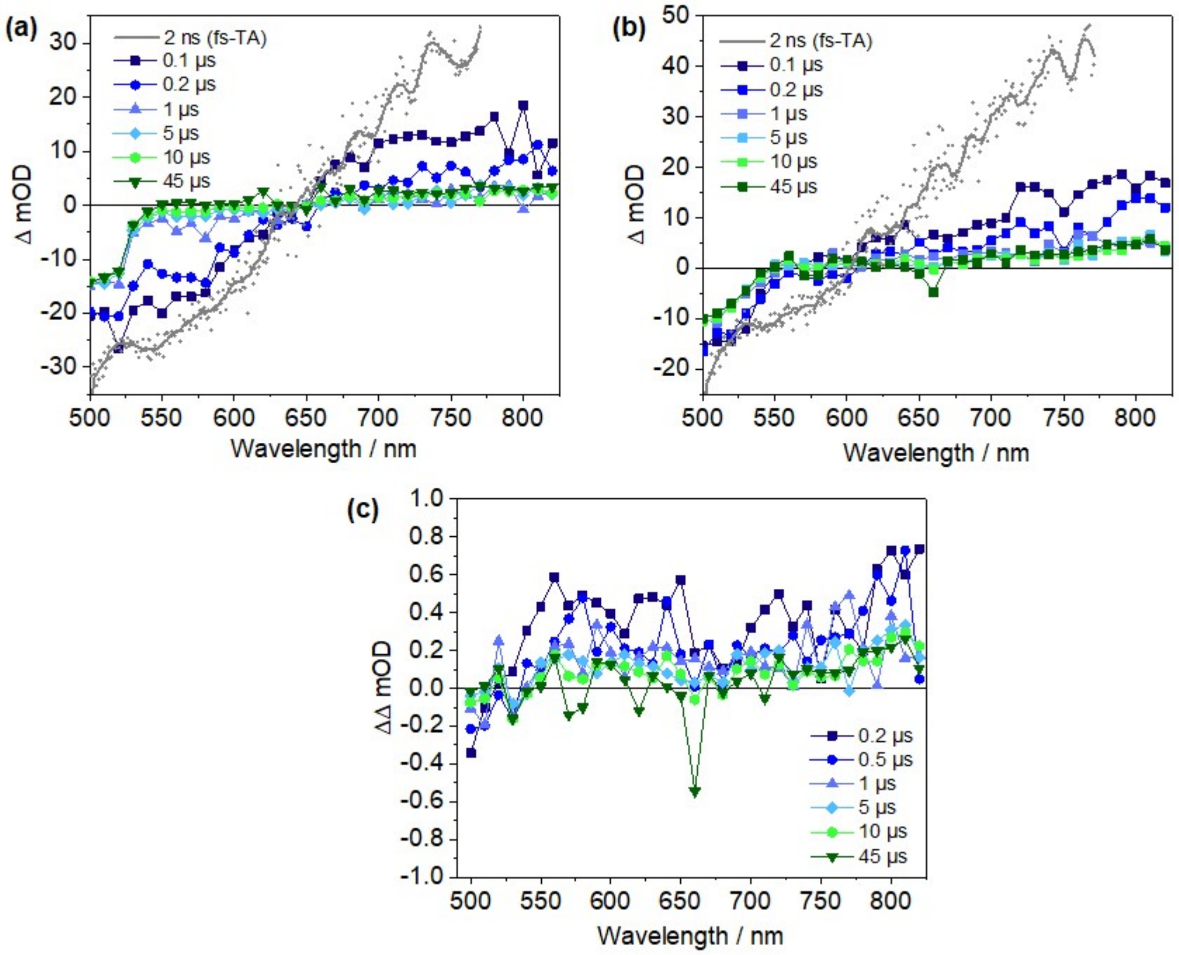

Figure S17. ns-transient absorption spectra of (a) **PPV** and (b) **PPV-Ru1** in $CHCl_3$ at different delay times. The pump pulses are centred at 480 nm. The grey spectra in panel (a) and (b) depict the fs transient absorption spectra at 2 ns, which is arbitrarily scaled to fit the ns-transient absorption data. (c) The differential ns-transient absorption spectra of **PPV-Ru1** (after PPV subtraction) at different delay times.

As we aim to develop **PPV-Ru** photocathodes, transient absorption spectroscopy study of films of **PPV**, **Ru**, and **PPV-Ru** is carried out. In general, transient absorption data of **PPV**, **Ru**, and **PPV-Ru** films is slightly different compared to the transient absorption data in solution. The transient absorption data of **PPV** film show an initial ground-state bleach (GSB), stimulated emission (SE) at 525 (shoulder), 575, and 625 nm (peak), and an excited-state absorption (ESA) band beyond 700 nm (Fig. S18a). Nonetheless, it should be noted that the negative ΔOD peak at 625 nm contains contribution from both stimulated emission of 0-0 transition and the ground state

bleach of the vibronic 0-1 transition.[3,9,10] A global fit of TA data using three decay components results in the decay-associated spectra shown in Fig. S18c. The spectral features associated with τ_1 show negative ΔOD signal at 450 – 570 nm reflecting the decay of the early relaxation originates from a strong coupling between electronic and vibrational states, and hence, the fast kinetic processes are attributed to delocalized exciton states (self-trapping, ~ 100 fs).[15] The spectral signature of τ_2 is marked with the decay of GSB, SE at 575 nm and GSB of vibronic 0-1 transition at 625 nm. Also, this process (τ_2 = 11 ps) is assigned to interchain or intrachain energy transfer, which typically falls on a characteristic timescale of tens of ps[10] and the process associated with τ_3 is assigned to exciton recombination.

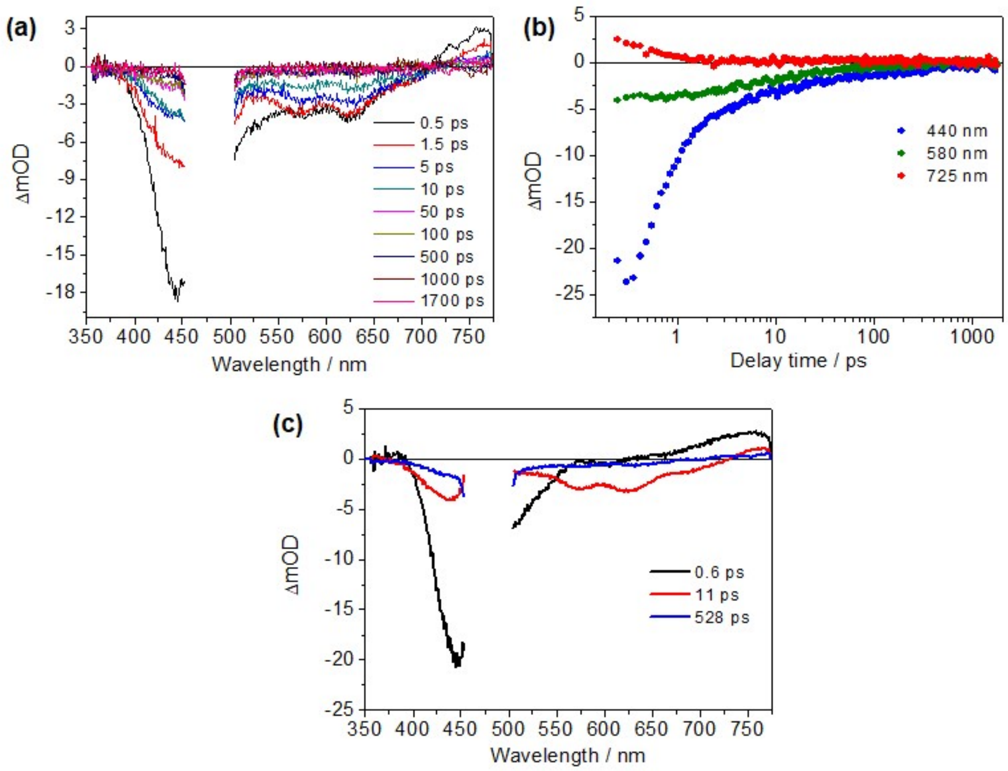

Figure S18. (a) fs-transient absorption spectra of **PPV** film at different delay times, (b) kinetic traces at different probe wavelengths, and (c) decay-associated spectra resulting from the global fit with three exponential decay function. The grey area depicts the corresponding inverted ground state absorption spectrum of **PPV**, which is arbitrarily scaled to fit the transient absorption data.

Similar to the measurement in solution, the transient absorption spectra of **Ru1** films exhibit a broad and rather unstructured ESA beyond 560 nm for **Ru1** due to ligand-to-metal charge transfer transitions (π-bpy → dπ) with a GSB centered at around 450 nm. The band at 385 nm for **Ru1** is assigned to the π–π* transition at the $bpy^{\bullet-}$ fragment.[11,12] Unlike transient absorption data in solution, the amplitude of the initial transient absorption signal decays by *ca.* 80% for both photosensitizers indicating that the ^{3}MLCT excited states typical for Ru tris-diimine complexes is short-lived in films. This short-lived ^{3}MLCT excited state can be due to oxygen quenching as the **Ru1** films are exposed to ambient air.[13,14] A quantitative analysis by globally fitting with two-exponential decay functions and an

infinite component results in characteristic time constants (τ_1 = 15 ps, τ_2 = 300 ps for **Ru1**) and decay-associated spectra shown in Fig. S19c.

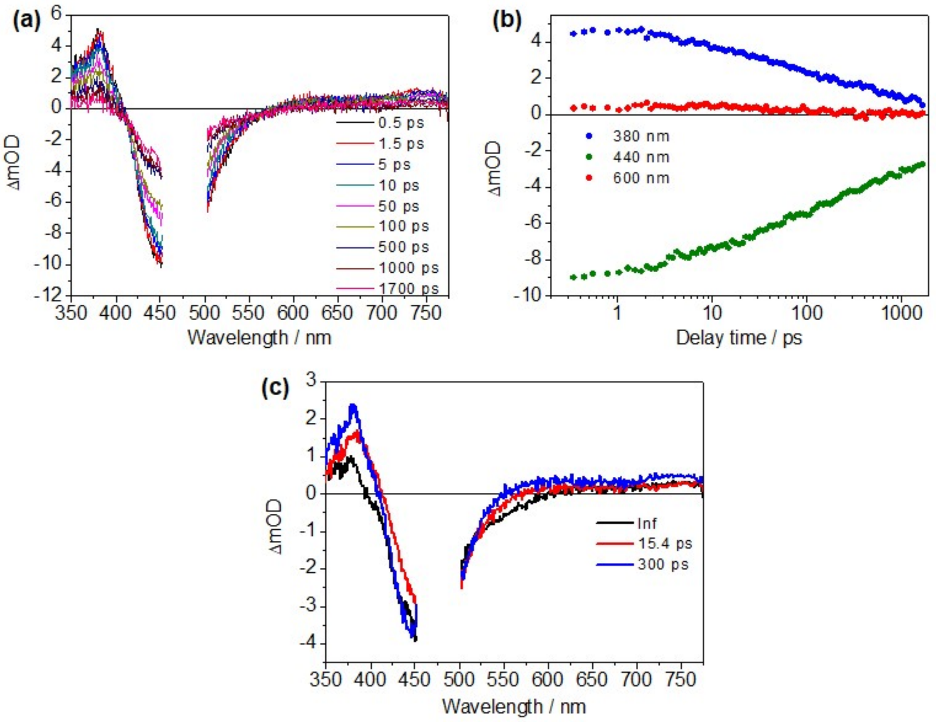

Figure S19. (a) fs-transient absorption spectra of **Ru1** film at different delay times, (b) kinetic traces at different probe wavelengths, and (c) decay-associated spectra resulting from the global fit with two exponential decay function and an infinite component compensating the long-lived signal.

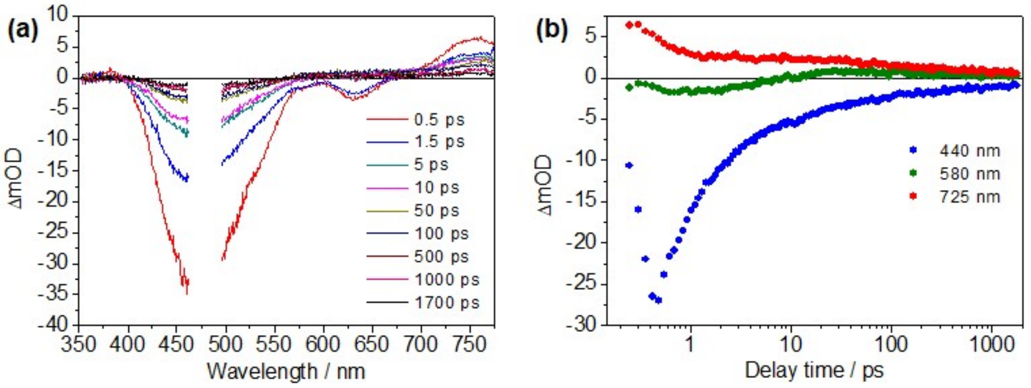

Figure S20. (a) fs-transient absorption spectra of **PPV-Ru1** film at different delay times and (b) kinetic traces at different probe wavelengths.

Similar to the transient absorption data in solution, the transient absorption spectra of **PPV-Ru1** (Figure S20c) are dominated by the spectral signature of **PPV** as the loading of the polymer with either of the photosensitizers is comparably low, *i.e.* 7.5% loading density. Figure S20 indicates that some differences in the transient absorption

spectra of **PPV-Ru1** are observed compared to the reference **PPV** data. The spectral shape at the red flank of the negative ΔOD differs slightly due to spectral overlap of GSB contribution from **PPV** and contributions from photosensitizer-associated excited-state absorption. Also the zero-crossing (ΔOD = 0) of **PPV-Ru1** shifts to a shorter wavelength at early delay time (Δt = 1.5 ps) as compared to the reference **PPV**, *i.e.*, from 708 to 670 nm for **PPV-Ru1**.

Similar to the transient absorption data processing for **PPV-Ru** in solution, in order to analyse the light-induced charge transfer process across the **PPV-Ru** film, the data obtained for **PPV** alone is subtracted from the transient absorption signal of **PPV-Ru**. Prior to subtraction the data is normalized to its early time (Δt = 5 ps) signal amplitude at 560 nm for **PPV-Ru1** at which there is no contribution of the transient absorption signal from **Ru** alone (see Figure S19a). Thus, similar to the analysis of TA data in solution, the subtraction allows us to unravel the excited state interactions between the photosensitizer and the **PPV** in the photoexcited **PPV-Ru** system. The subtracted, *i.e.* differential transient absorption spectra, *i.e.*, $\Delta\Delta OD = \Delta OD_{[PPV\text{-}Ru]} - \Delta OD_{[PPV]}$, is determined as follow.

$$\Delta\Delta OD(\Delta t,\lambda) = \frac{\Delta OD(\Delta t,\lambda)_{[PPV-Ru]}}{\Delta OD(\Delta t = 5ps, \lambda = 560nm)_{[PPV-Ru]}} - \frac{\Delta OD(\Delta t,\lambda)_{[PPV]}}{\Delta OD(\Delta t = 5ps, \lambda = 560nm)_{[PPV]}}$$

The ΔΔOD spectra are subjected to a global analysis using a multi-exponential fit. The decay-associated spectra (DAS) generated from a fit function consisting of a sum of three exponentials for **PPV-Ru1** are shown in Figure S21.

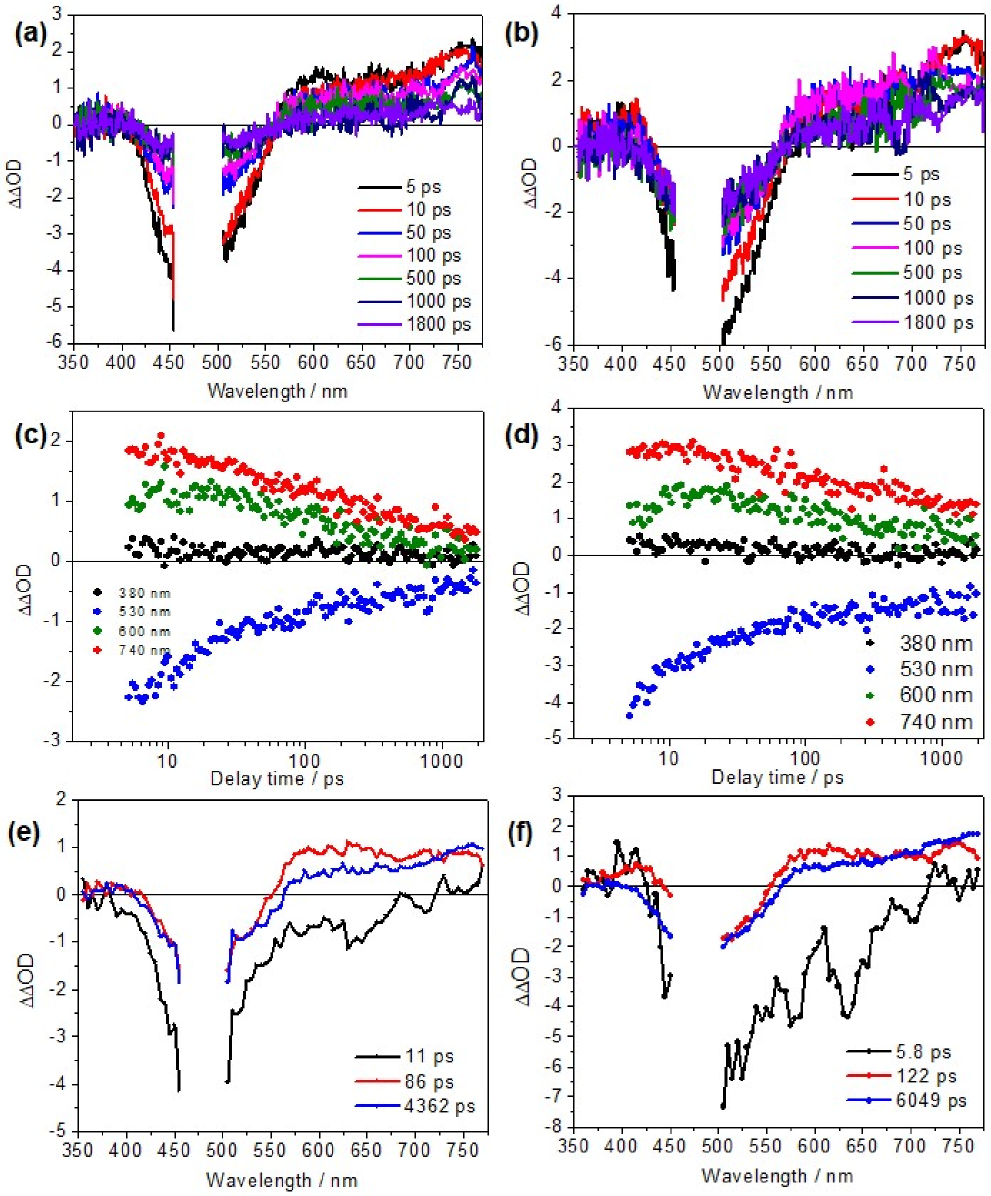

Figure S21. Differential fs-transient absorption spectra of **PPV-Ru1** film at different delay times (a,b). Kinetic traces at different probe wavelengths (c,d) and decay-associated spectra (DAS) (e,f) resulting from the global fit with two time constants for **PPV-Ru1**. The pump pulses are centred at 480 nm.

Table S2. Characteristic time constants (τ) from global fitting of differential fs-transient absorption data of **PPV-Ru** films from three different measurements.

System	τ_1 / ps	τ_2 / ps	τ_3 / ps
**PPV-Ru1**	8.3 ± 2.6	106 ± 19	4841 ± 1052

5. Photoelectrochemical Measurements

It is already discussed in the main manuscript the linear scan voltammogram of the drop-casted films of **PPV**, **PPV-Ru1** and **PPV-Ru2**, in which the photocurrent density is normalized to the maximum absorbance. Here is the original photocurrent density response obtained in the linear scan voltammogram.

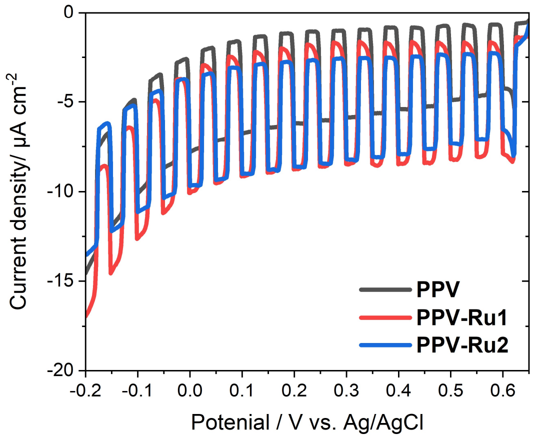

Figure S22. Linear scan voltammogram (scan speed 20 mV/s) of drop-casted films of **PPV**, **PPV-Ru1** and **PPV-Ru2** in Co^{III}/Co^{II} electrolyte under chopped illumination (1000 nm > λ > 300 nm, 1000 W·m^{-2}) divided by the film surface area to obtain current densities. Shown are the average values of four individual measurements.

6. Photostability Test of PPV-Ru System

To check the photostability of each component in PPV-Ru system during photoelectrochemical and transient absorption measurements, pre- and post-operando UV/vis absorption spectroscopy were carried out. The absorption spectra for **PPV**, **PPVRu1**, and **PPVRu2** films were measured both before and after the chopped-light voltammetry (CLV, 10 min irradiation) and linear scan voltammetry (LSV, 1-2 min irradiation) experiments in the SEC cuvette without the electrolyte solution.

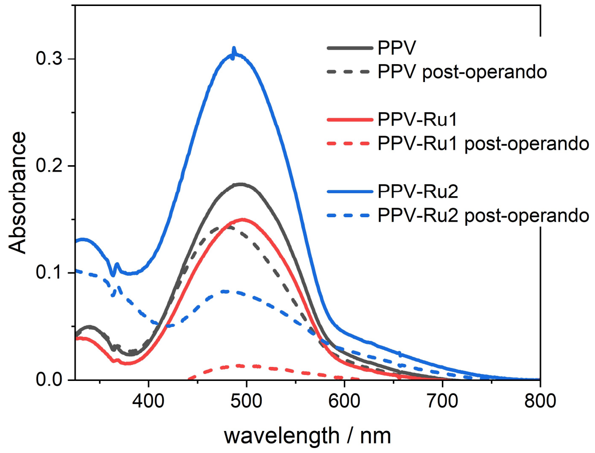

Figure S23. UV/vis spectra of drop-casted films of **PPV**, **PPV-Ru1** and **PPV-Ru2** as prepared (solid) and after the photoelectrochemical experiments (LSV and chronoamperometry under chopped irradiation, dashed lines).

To probe the photostability of Co^{III}/Co^{II} electrolyte, the absorption spectra of the electrolyte solution in the SEC cuvette without the electrodes were also measured before and after irradiation in both the CLV and LSV experiments. The results indicate that the electrolyte seemed to be stable in the relatively short time of the photoelectrochemical experiments as no changes were observed in the UV/vis spectra recorded pre- and post-operando (see Fig. S24).

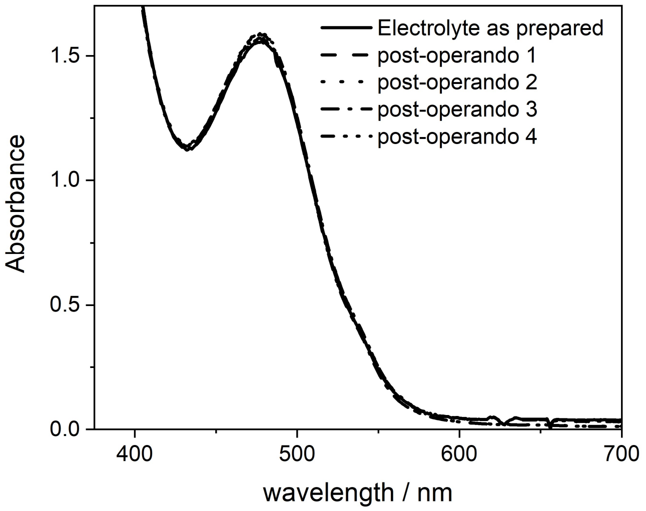

Figure S24. Pre- and post-operando UV/vis spectra of the electrolyte used for the photoelectrochemical experiments.

To ensure that the data obtained from fs-transient absorption spectroscopy is reliable for analysis, here we show the UV/vis absorption spectra of **Ru1**, **PPV**, and **PPV-Ru1**. It should be noted that the sample solution as well as the film was moved during the scan allowing the pump-probe beam in the transient absorption measurement to hit the fresh sample area and hence, signals from the non-photodegraded sample volume were collected. As shown in Fig. S25, no change in absorption spectrum is observed for **Ru1.** However, a slight decay of absorption signal is observed for both **PPV** and **PPV-Ru1** after fs-transient absorption measurement. The results already indicate the photo-instability of the **PPV** backbone.

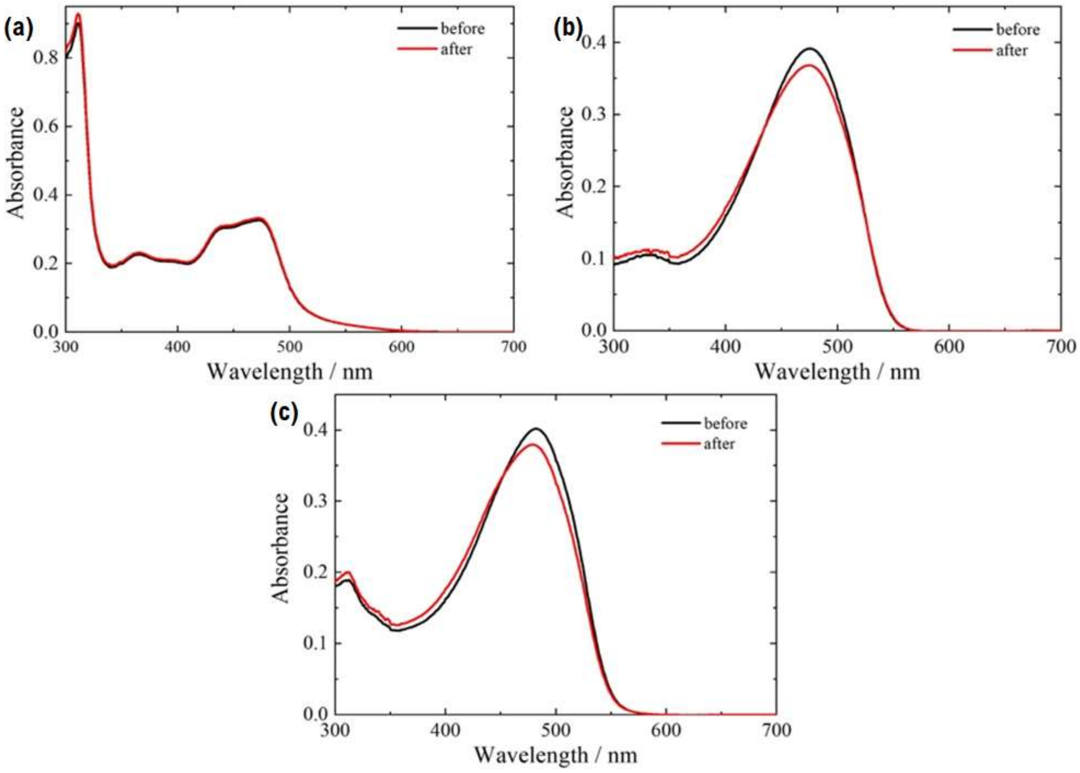

Figure S25. UV/vis spectra of (a) **Ru1**, (b) **PPV** and (c) **PPVRu1** (7% loading) measured before and after fs-transient absorption spectroscopy measurement.

References

1. Wintergerst, P; Mengele, A.; Nauroozi, D.; Tschierlei, S.; Rau, S., Impact of Alkyne Functionalization on Photophysical and Electrochemical Properties of 1,10-Phenanthrolines and Their Ru^{II} Complexes. *Eur. J. Inorg. Chem.* **2019**, 1988–1992.
2. Kowacs, T.; Pan, Q.; Huiiser, A.; Rau, S.; Lang, P.; Browne, W. R.; Pryce, M. T.; Vos, J. G., Supramolecular bimetallic assemblies for photocatalytic hydrogen generation from water, *Inorg. Chem.* **2016**, 55, 2685-2690.
3. Consani, C.; Koch, F.; Panzer, F.; Unger, T.; Köhler, A.; Brixner, T. Relaxation dynamics and exciton energy transfer in the low temperature phase of MEH-PPV. *J. Chem. Phys.* **2015**, 142, 212429.
4. Meskers, S. C. J.; Janssen, R. A. J.; Haverkort, J. E. M.; Wolter, J. H. Relaxation of photo-excitations in films of oligo- and poly-(para-phenylene vinylene) derivatives. *Chem. Phys.* **2000**, 260, 415.
5. Herz, L. M.; Silva, C.; Grimsdale, A. C.; Müllen, K.; Phillips, R. T. Time-dependent energy transfer rates in a conjugated polymer guest-host system. *Phys. Rev. B* **2004**, 70, 165207
6. Wang, J.; Price, R. S.; Leem, G.; Jiang, J.; Abboud, K. A.; Schanze, K. S. Interaction of a Poly(phenylene-vinylene) with an Organometallic Lewis Acid Additive: Fundamentals and Application in Polymer Solar Cells. *Chem. Mater.* **2018**, 30, 596-5977.
7. Burrows, H.D.; Miguel, M. da G.; Monkman, A.P.; Hamblett, I.; Navaratnam, S. Transient absorption spectra of triplet states and charge carriers of conjugated polymer. *J. Mol. Struct.* **2001**, 563-564, 41-50;
8. Monkman, A.P.; Burrows, H.D.; Miguel, M. da G.; Hamblett, I.; Navaratnam, S. Triplet state spectroscopy of conjugated polymers studied by pulse radiolysis. *Synthetic Metals* **2001**, 116, 75-79.
9. Lim, S.-H.; Bjorklund, T. G.; Bardeen, C. J. Temperature-dependent exciton dynamics in poly(p-phenylene-vinylene) measured by femtosecond transient spectroscopy. *Chem. Phys. Lett.* **2001**, 342, 555-562.
10. Yu, Z.; Barbara, P. F. Low-Temperature Single-Molecule Spectroscopy of MEH-PPV Conjugated Polymer Molecules. *J. Phys. Chem. B* **2004**, 108, 11321-11326.
11. Vandewal, K.; Benduhn, J.; Nikolis, V. C. How to determine optical gaps and voltage losses in organic photovoltaic materials. *Sustainable Energy Fuels* **2018**, 2, 538.
12. Sun, Y.; Chen, Z.; Puodziukynaite, E.; Jenkins, D. M.; Reynolds, J. R.; Schanze, K. S. Light harvesting arrays of polypyridine ruthenium(II) chromophores prepared by Reversible Addition-Fragmentation Chain Transfer polymerization. *Macromolecules* **2012**, 45, 2632.
13. Draxler, S.; Lippitsch, M. E.; Klimant, I.; Kraus, H.; Wolfbeis, O. S. Effect of Polymer Matrices on the Time-Resolved Luminescence of Ruthenium Complex Quenched by Oxygen. *J. Phys. Chem.* **1995**, 99, 3162-3167.
14. Hartmann, P.; Leiner, M. J. P. Luminescence Quenching Behavior of an Oxygen Sensor Based on a Ru(II) Complex Dissolved in Polystyrene. *Anal. Chem.* **1995**, 67, 88-93.
